# The prevalence of gene duplications and their ancient origin in *Rhodobacter sphaeroides *2.4.1

**DOI:** 10.1186/1471-2180-10-331

**Published:** 2010-12-30

**Authors:** Anish Bavishi, Lin Lin, Kristen Schroeder, Anne Peters, Hyuk Cho, Madhusudan Choudhary

**Affiliations:** 1 Department of Biological Sciences, Sam Houston State University, Huntsville, Texas, 77341, USA; 2 Department of Computer Science, Sam Houston State University, Huntsville, Texas, 77341, USA

## Abstract

**Background:**

*Rhodobacter sphaeroides *2.4.1 is a metabolically versatile organism that belongs to α-3 subdivision of *Proteobacteria*. The present study was to identify the extent, history, and role of gene duplications in *R. sphaeroides *2.4.1, an organism that possesses two chromosomes.

**Results:**

A protein similarity search (BLASTP) identified 1247 *orfs *(~29.4% of the total protein coding *orfs*) that are present in 2 or more copies, 37.5% (234 gene-pairs) of which exist in duplicate copies. The distribution of the duplicate gene-pairs in all Clusters of Orthologous Groups (COGs) differed significantly when compared to the COG distribution across the whole genome. Location plots revealed clusters of gene duplications that possessed the same COG classification. Phylogenetic analyses were performed to determine a tree topology predicting either a Type-A or Type-B phylogenetic relationship. A Type-A phylogenetic relationship shows that a copy of the protein-pair matches more with an ortholog from a species closely related to *R. sphaeroides *while a Type-B relationship predicts the highest match between both copies of the *R. sphaeroides *protein-pair. The results revealed that ~77% of the proteins exhibited a Type-A phylogenetic relationship demonstrating the ancient origin of these gene duplications. Additional analyses on three other strains of *R. sphaeroides *revealed varying levels of gene loss and retention in these strains. Also, analyses on common gene pairs among the four strains revealed that these genes experience similar functional constraints and undergo purifying selection.

**Conclusions:**

Although the results suggest that the level of gene duplication in organisms with complex genome structuring (more than one chromosome) seems to be not markedly different from that in organisms with only a single chromosome, these duplications may have aided in genome reorganization in this group of eubacteria prior to the formation of *R. sphaeroides *as gene duplications involved in specialized functions might have contributed to complex genomic development.

## Background

*Rhodobacter sphaeroides *2.4.1, a purple nonsulfur photosynthetic eubacterium, belongs to the α-3 subgroup of *Proteobacteria *[1,2], members of which display an array of metabolic capabilities in the assembly and regulation of metabolic functions [3], electron transport [4-6], bioremediation [7], and tetrapyrrole biosynthesis [8,9]. In addition, many members of this subgroup establish different types of eukaryotic associations [10-14]. The genome of *R. sphaeroides *2.4.1 has been completely sequenced and annotated [15] and is comprised of two circular chromosomes and five plasmids.

Bacterial species continue to encounter different ecological niches, and their genome size increases by acquiring habitat relevant genes by horizontal gene transfer [16-18] and gene duplication [19,20], which together play a major role in the evolution of both genome size and complexity. Duplicated genes are ubiquitously present among eukaryotes and prokaryotes [21-24]. Analyses on over 100 fully sequenced eubacterial and archaeal genomes have revealed a great extent of DNA sequence duplications [25], however it remains unclear whether the expansions of genome size and complexity were essential for adaptive phenotypic diversification.

The present study aimed to systemically identify the extent and history of gene duplication in the genome of *R. sphaeroides*. A hypothesis that the complex genome structure (large genome size and the presence of multiple chromosomes) requires an extensive amount of gene duplications was examined by determining the distribution of duplicated genes on both chromosomes and plasmids and comparing the determined levels of *R. sphaeroides *gene duplication to that in other bacterial species that possess a single chromosome. After determining the extent of these gene duplications, two additional hypotheses were devised. First, a hypothesis was formulated to test whether gene duplications were selectively preserved in specific Clusters of Orthologous Groups (COGs) necessary to accommodate the diverse growth mode of this organism. Second, a hypothesis was tested to ascertain whether this level of large-scale gene duplications occurred after the diversification of members of the α-3 subgroup of *Proteobacteria*. The role of gene duplications in understanding the evolution of new metabolic functions is discussed along with the age and functional constraints of these gene pairs across four strains of *R. sphaeroides*. Thus, this study investigates the nature of gene duplications in an organism with complex genome structuring in order to determine the role of such duplications in the evolution of new metabolic functions and complex genome development.

## Methods

### Protein homology and duplication search analysis

A protein homology search was performed using the gapped BLASTP [26], which included gap penalties, and was therefore more conservative in database searches. The search was conducted in two steps. First, each protein sequence of the *R. sphaeroides *genome was used to search the homologous proteins against their own database. Then, each of the corresponding homologous protein sequences identified by the first step was reciprocally paired, based on a threshold E-value of ≤ 10^-20^. The cut-off value for the percent amino acid identity was set at ≥ 30%, which defines the level above which gene duplication can be reliably identified in many bacterial species [15,27,28]. However, certain duplicated genes in *R. sphaeroides *that did not meet the specified search criteria (i.e. possessed less than 30% identity) have been identified or reported in the past [15,28]. These identified or reported duplications were incorporated for subsequent analysis. Also, to approximately determine the prevalence and arrangement of selected gene duplications in three other completely sequenced *R. sphaeroides *strains (ATCC 17025, ATCC 17029, KD131), each gene (those designated as "Orf 1") in a duplicated pair in *R. sphaeroides *2.4.1 was subjected to BLASTP analysis against the three *R. sphaeroides *strains, with the same cutoff criteria utilized as before.

### Analysis of the Cluster of Orthologous Groups (COGs)

Gene homologs are families of genes, which encode similar protein functions within a genome and between genomes; if such genes are derived from different species, they are called orthologs, and if they are derived from the same species, they are referred to as paralogs [29]. The Cluster of Orthologous Groups [30,31] classifications provide a tool in examining gene roles. There are four major COG functions, which include 1: Information storage and Processing, 2: Cellular Processes, 3: Metabolism, 4: Poorly Characterized functions. These major groupings were further classified into 25 sub-groups. However, a number of Orfs have been classified into more than one COG as they encode overlapping gene functions, while other Orfs have poorly characterized functions. The percentage of each COG functions, both in the general groups and the sub-groups, among the duplicated genes was compared with the percentage of the respective COG functions over all genes present in the complete genome. A chi-square (χ^2^) test was performed for both distribution comparisons with a null hypothesis assuming that the gene duplications have the same COG distributions as all the genes in the full genome. In addition, all 234 pairs were subsequently mapped onto CI and CII. The level of divergence was indicated by the y-axis and the height of the gene pinning and each gene's major COG group classification was color-coded.

### Phylogenetic Analysis

To determine the origin and history of the gene duplications in *R. sphaeroides*, initially each protein in the protein-pairs was blasted against the microbial database at NCBI using the BLASTP [26]. Geneious v4.6, a versatile bioinformatics suite, was used to organize and perform the protein similarity searches, generate alignments, and construct phylogenetic trees [32]. Only organisms with completely sequenced genomes were chosen to avoid poor or incomplete sequence data from shotgun or partial genome sequencing projects.

For each set of homologous matches, there were four proteins: the duplicated genes and an ortholog match for each copy as only the best and most complete hits to each gene in a pair were selected. For these duplicate pairs, two alternative phylogenetic relationships were predicted. The Type-A relationship was predicted when a protein sequence branched with a homolog (ortholog) from a closely related species rather than its counterpart protein (paralog) within the *R. sphaeroides *genome, whereas as Type-B relationship was predicted when the duplicate protein copies within *R. sphaeroides *branched with each other [28,33]. Additionally, four example phylogenetic analyses, two exhibiting Type-A phylogeny and two exhibiting Type-B phylogeny, were carried out with gene duplications common among the four *R. sphaeroides *strains.

Protein sequence alignments were carried out using MUSCLE [34], a program known for its accuracy and speed. Phylogenetic analysis was performed using PhyML [35] with the WAG model [36] to generate unrooted, maximum likelihood trees. Bootstrap values were calculated using 100 replications for the trees where topology was being determined. Maximum likelihood trees were constructed for all protein-pairs to ascertain the tree topology (Type-A or Type B). If a set of duplicated genes had their highest match to the same ortholog, then the next highest ortholog match, if available, for one of the genes was utilized in the tree construction to ascertain accurately the duplication topology.

### Functional Constraints Analysis

For the functional constraints analysis, comparisons were conducted within all four *R. sphaeroides *strains. More specifically, the 28 common gene pairs among the four strains were utilized for the functional constraints analysis where the genes in a given pair were compared against one another. The synonymous and nonsynonymous substitution rates along with the nonsynonymous-synonymous substitution rate ratio were calculated using the modified Yang-Nielsen algorithm [37,38]. MUSCLE was used to align amino acid sequences [34]. These aligned sequences were then transformed into the original DNA sequences after which, the K_a_K_s__Calculator was used with each pair of DNA sequences [39] to calculate the synonymous substitution rate (K_s_), the nonsynonymous substitution rate (K_a_), and the nonsynonymous/synonymous rate ratio (ω = K_a_/K_s_). Under the MYN model, ω = 0.3, 1, and 3 were used for negative (purifying), neutral, and positive selection, respectively [37,38]. A one-way ANOVA was used to test whether the distributions of ω among the four strains were dissimilar.

### Horizontal Gene Transfer

Horizontal gene transfer (HGT) features were estimated using Alien-Hunter, which predicts HGT events using interpolated variable order motifs [40]. This method exploits compositional biases to determine potential HGT areas where abnormal (HGT) areas are identified as those that are higher than a threshold value, a value that is calculated using the sequence structure of the input genome among other factors. This software was used to determine the areas of possible HGT and the levels of HGT on CI and CII independently. The genes present within these regions were additionally identified. Artemis [41] was used to view the Alien-Hunter output.

## Results

### Extent of gene duplications in *R. sphaeroides*

Of the total 4242 protein coding genes in its genome, a total of 1247 genes (29.4% of its genome) exist in multiple copies in the *R. sphaeroides *genome. Gene homologs are present in different copies reflecting the diversity of gene multiplication. Numbers of genes with 2, 3, 4 and 5 and more (≥ 5) copies were 468, 183, 152, and 444, respectively. Approximately 73% of the total gene homologs represent two classes, genes with two copies (37.5%; 234 protein pairs) and genes with ≥ 5 copies (35.6%). Genes with ≥ 5 copies represent various types of functions, for example, ABC type transporters, families of transcriptional factors, and cell-signaling response regulators (data not shown). If genes that are present in more than two copies were to be selected, determining the lineage of such genes becomes functionally more complex, especially as many such genes are also present within multiple gene families. Moreover, the genes in these families can be analogous instead of homologous, meaning that they are similar due to function rather than origin. As such, further analysis was carried out only on genes which were identified as duplicate protein pairs as listed in Additional file 1.

**Figure 1:**
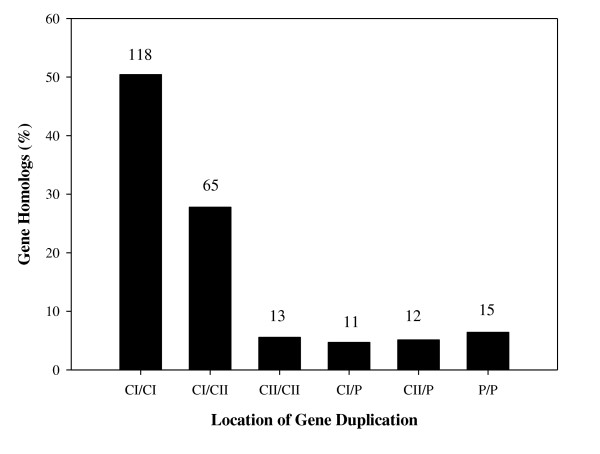
**A distribution of the gene duplications based on their location in the genome**. The number of duplicate gene-pairs present in each group is given on top of the bars while the y-axis specifies the percentage that each group makes up of all duplicate gene pairs. (CI: Chromosome I; CII: Chromosome II; P: Plasmids)

The mean amino acid identity of the protein-pairs was 46.0% and the standard deviation was 19.5% with a maximum amino acid identity of 99%. Gene homologs are dispersed either within each replicon or between replicons in the genome of *R. sphaeroides *as shown in Figure 1. Of the total 234 duplicate-genes, 196 gene duplications (83.8%) were chromosomal and 38 gene duplications (16.2%) were dispersed between chromosome and plasmid or between plasmids. Of chromosomal gene duplications, intra-chromosomal and inter-chromosomal gene duplications were 131 (56.0%) and 65 (27.8%), respectively. Of the 131 intra-chromosomal gene duplications, 118 (50.4%) and 13 (5.5%) gene homologs were located within CI and CII, respectively. Taking the sizes of the two chromosomes into account (CI is three times larger than the size of CII); the number of gene duplications found within CI was significantly higher than the number of gene duplications found within CII. Approximately 16.2% of gene duplications involve plasmids where 9.8% of the total gene duplications involve plasmids and chromosomes while 6.4% of the total genes duplications were solely between plasmids.

**Figure 2:**
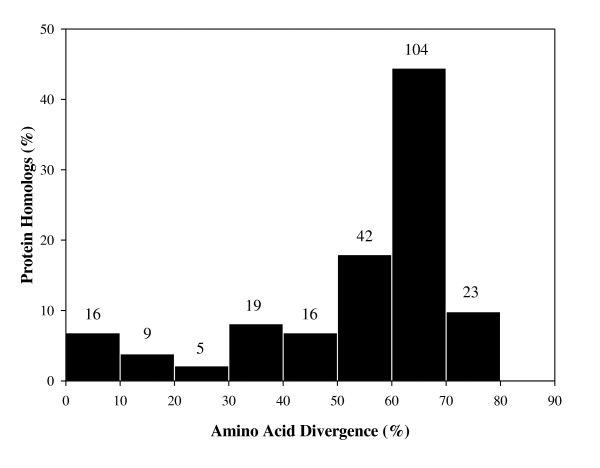
**A distribution of the two duplicate protein pairs based on the percent amino acid divergence**. The number of duplicate protein-pairs present for each divergence group is given on top of the bars while the y-axis represents the percentage that each group makes up of all of the duplicated protein pairs.

The relationship between the percentage of homologous gene-pairs and their corresponding level of amino acid divergence is shown in Figure 2. Amino acid divergence is defined as 100% minus the percentage identity between the protein sequences. The protein sequence conservation of the duplicated protein pairs varied widely. Of the 234 gene-pairs, 204 gene-pairs showed ≥30% amino acid divergence between their corresponding protein homologs reflecting the rapid evolution of these proteins, while 30 protein-pairs demonstrated <30% divergence. Forty-two protein-pairs (17.9%) have diverged between 51% - 60% of their of protein sequences, 104 pairs (44.4%) exhibit the amino acid divergence ranging from 61% - 70%, and approximately 10% (23 protein-pairs) of the total protein-pairs displayed amino acid divergence between 71%-80%. A majority of gene homologs with low divergence (< 30%) were representative of essential functions, of which 16 protein-pairs are conserved hypothetical proteins whose metabolic functions remain unknown. The more conserved proteins included for instance, DNA binding proteins (ParA, ParB, Spb, a histone-like protein, cold-shock DNA binding proteins), chemotaxis response regulators (CheY), and periplasmic serine proteases (ClpP, ClpX). On the other hand, gene homologs with high level of amino divergence represented proteins involved in cell structure (flagella formation) and cellular processes like metabolism, transport, replication, transcription (σ factors), and translation (see Additional file 1 for more information).

### Gene duplication and diverse COGs functions

**Figure 3:**
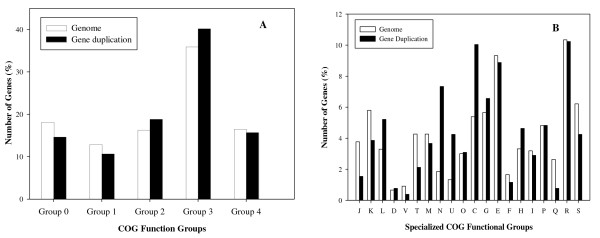
**A. A distribution of the two copy genes based on general Clusters of Orthologous Groups of proteins (COG) functions**. The genes are classified in 5 generalized groups: Not in COGs (Group 0); Information storage and processing (Group 1); Cellular processes (Group 2); Metabolism (Group 3); Poorly characterized (Group 4). **B**. A distribution of the two copy genes based on specific Clusters of Orthologous Groups (COGs) of protein functions. A more detailed breakdown of the distribution of the genes is given based on different cellular functions represented in 25 COG sub-groups. Of these classifiable COG groups, duplicated genes are present in 20 subgroups: J. Translation, ribosomal structure and biogenesis; K. Transcription; L. DNA replication, recombination and repair; D. Cell division and chromosome partitioning; V. Defense mechanisms; T. Signal transduction mechanisms; M. Cell envelope biogenesis, outer membrane; N. Cell motility and secretion; U. Intracellular trafficking and secretion; O. Posttranslational modification, protein turnover, chaperones. C. Energy production and conversion; G. Carbohydrate transport and metabolism; E. Amino acid transport and metabolism; F. Nucleotide transport and metabolism; H. Coenzyme metabolism; I. Lipid metabolism; P. Inorganic ion transport and metabolism; Q. Secondary metabolites biosynthesis, transport and catabolism; R. General function prediction only; S. Function unknown.

The distribution of the duplicated genes present in each of the cluster of orthologous group (COGs) was compared to distribution of genes representing these general COGs in the complete genome as shown in Figure 3A. Gene duplications were represented by all the COGs, which included information processing (COG 1), cellular processing (COG 2), metabolism (COG 3), and poorly characterized functions (COG 4). A number of gene duplications were not yet classified in any of these COG functions (COG 0) since their functions are currently unknown. For these analyses the individual genes were examined since the copies have diverged in function from their ancestors. For protein-pairs with multiple functions, the COGs were counted by their categorizations, although this was a relatively infrequent occurrence (8 genes). Analysis showed that the gene duplications representing information processing, cellular processes, metabolism, and poorly characterized functions constituted 10.7%, 18.8%, 40.2%, and 15.7% of the gene duplications, respectively. The percentage of genes in the genome of *R. sphaeroides *that fell under these general COG categories of information processing, cellular processes, metabolism, and poorly characterized were 12.9%, 16.3%, 36.0% and 16.5%, respectively (data taken from NCBI). The chi-square analysis demonstrated that the proportion of duplicated genes involved in metabolism, information processing, cellular processes, or unknown functions were significantly different from the overall proportion of total genes representing these functions present in the complete genome (χ^2 ^value = 9.585, *p *< 0.05). Further analysis on more specific COGs revealed a greater distribution difference between the gene duplications and the genes in the total genome, as shown in Figure 3B. A chi-square test confirmed that the distributions were significantly different (χ^2 ^value = 175.5041, *p *< 0.0001). The analysis revealed that genes involved in group L (DNA replication, recombination and repair), group N (cell motility and secretion), group U (intracellular trafficking and secretion), group C (energy production and conversion), group G (carbohydrate transport and metabolism), and group H (coenzyme metabolism) were overrepresented among genes evolved by gene duplication, while number of genes representing other COG subgroups remained low or fairly equal in percentages to the number of genes representing those COGs in the overall genome of *R. sphaeroides*.

**Figure 4:**
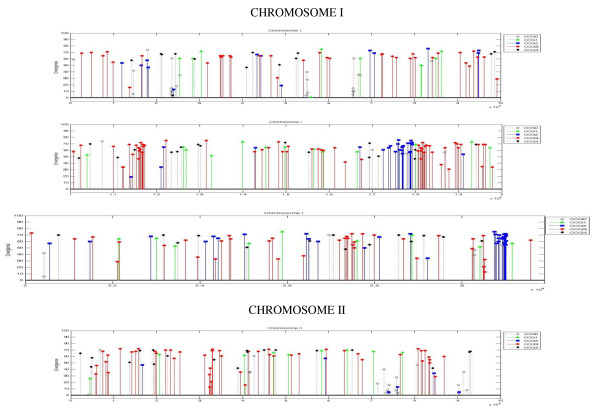
**Location of gene duplications on chromosome I and II**. These plots depict the distribution of the 234 duplicate pairs across CI and CII. The y-axis represents the level of divergence for a gene in a pair and the genes are color-coded to represent their COG function grouping. The plots reveal several clusters of gene duplications of similar COG function on CI and CII.

Figure 4 depicts the distribution of the gene duplications on CI and CII. Although the majority of gene duplications seem to be randomly distributed, there are a few locations where clusters of gene duplications that possess similar COG functions are found. On CI, duplicated gene clusters representing COG 2 (cellular processes) were found at two locations: between 1.7 - 1.8 Mb and between 3.0 - 3.1 Mb. In addition, duplicated gene clusters representing COG 3 (metabolism) were uncovered between 1.1 - 1.2 Mb and between 1.8 - 1.9 Mb. On CII, two duplicated gene clusters representing COG 3 were present between 0.3 - 0.4 Mb and between 0.8 - 0.9 Mb. In addition, most of the gene duplications in these clusters exhibit roughly the same level of amino acid divergence.

**Figure 5:**
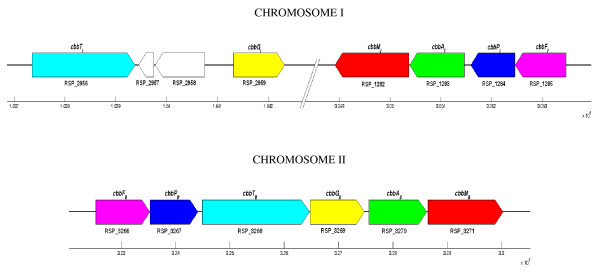
**Distribution of carbon metabolism gene duplications on chromosome I and II**. Only those with filled colors are carbon metabolism genes and the paired colors represent a given duplicate gene pair. Two clusters on CI contains carbon metabolism genes, while the duplicate gene counterparts are present in one cluster on CII.

Also, as about 40% of the gene duplications in *R. sphaeroides *2.4.1 are involved in cellular metabolism, it is important to analyze some specific components of gene duplication as related to cellular metabolism. Carbon fixation is an important metabolic pathway that contributes towards primary productivity and the physiological significance of carbon fixation in α-*Proteobacteria *species, including *R. sphaeroides*, is poorly understood. However, a distinct organization of gene duplications representing carbon metabolism is present in *R. sphaeroides*. As shown in Figure 5 there are two gene clusters on CI containing *cbbA*, *cbbF*, *cbbG*, *cbbM*, *cbbP*, and *cbbT *while their duplicate counterparts exist in a single cluster on CII. The amino acid identities between these genes and their homologs on CII are 79% (*cbbA*), 68% (*cbbF*), 84% (*cbbG*), 31% (*cbbM*), 87% (*cbbP*), and 58% (*cbbT*). These gene clusters also seem to be well conserved among all four sequenced strains *R. sphaeroides *(2.4.1, ATCC 17025, ATCC 17029, and KD131).

### Origin of gene duplications and relationship among *R. sphaeroides *strains

**Figure 6:**
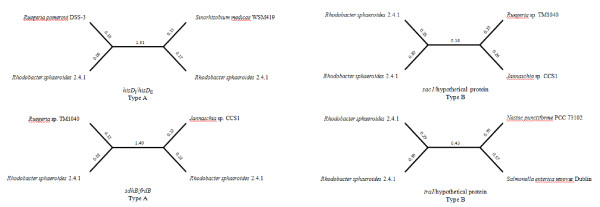
**The phylogenetic relationship of duplicate protein pairs and their highest matching ortholog sequences**. Maximum likelihood trees representing four of these relationships are shown above for *hisD_I _*and *hisD_II_*, *sdhB *and *frdB*, *sac1 *and a hypothetical protein, and *traI *and a hypothetical protein. Each of these unrooted trees displayed a bootstrap value of 100. The offshoots represent branches and their lengths are given (trees are not to scale). The relationships depict two types of topology - Type A or Type B.

As a sample, four phylogenetic trees, two of Type-A and two of Type-B, are shown in Figure 6. These phylogenetic trees depict data for *hisD*_I _and *hisD*_II_, *sdhB *and *frdB*, *sac1 *and a hypothetical gene, and *traI *and a hypothetical gene. Additional file 2 describes the amino acid identity for each duplicate protein-pair, the two ortholog matches, the identity with each of those ortholog matches to each *R. sphaeroides *protein in each duplicate protein-pair, the tree type (Type-A or Type-B) for the protein-pair, and the bootstrap values for each tree. Of the total 234 protein-pairs, ~77% of the protein-pairs (180 pairs) exhibited a Type-A relationship and ~23% of the protein-pairs (54 pairs) showed a Type-B relationship.

The strength of the tree topology was analyzed using bootstrap values, information concerning which is also shown in Additional file 2. Bootstrap values for 8 trees could not be determined due to the lack of one or more orthologs. Bootstrap values not only signify the significance of a tree topology (Type-A and Type-B), but also provide an insight into the relative origin of a given gene duplication. Gene duplication events that occurred significantly before organism speciation would display Type-A relationships with high bootstrap values. Gene duplication events that occurred significantly after organism speciation would display Type-B relationships with similarly high bootstrap values. Of the 226 trees for which bootstrap values were obtained, 209 (92.5%) had bootstrap values ≥ 95. The bootstrap values remained significant within both Type-A and Type-B phylogenetic trees. Of the 180 Type-A trees, 172 (95.56%) exhibited ≥ 95 bootstrap values while of the 46 Type-B trees, 37 (80.43%) exhibited ≥ 95 bootstrap values. Thus, the majority of these trees demonstrated correct and significant trees topologies, which support the relative timings of the origins of these gene duplications.

**Figure 7:**
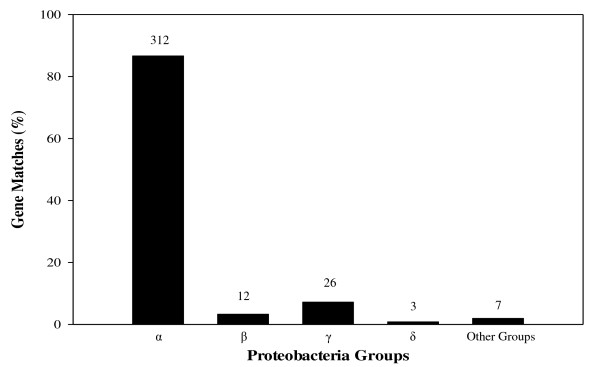
**Distribution of the highest ortholog matches for Type-A gene duplication matches**. The *Proteobacteria *groups are abbreviated to their subdivision. The amount of proteins in each group is shown on top of the columns while the y-axis depicts the percentage of the total amount that each column constitutes.

These results clearly show that a majority of gene duplications in *R. sphaeroides *originated prior to the formation of the *R. sphaeroides *lineage as also shown in Table 1. Of the Type-A gene duplications, 58.33% (105 pairs) were found only on CI, 26.67% (48 pairs) were found between CI and CII, and 6.11% (11 pairs) were found only on CII. Since about 91% of the duplications exhibiting a Type-A relationship were distributed on the two chromosomes, these results submit that the origin of multiple chromosomes in *R. sphaeroides *predates the origin of this species. 13 proteins had indiscernible matches to any orthologs in the current microbial database. Moreover, although a vast majority of the genes (312 of 360 genes, 86.67%) showing Type-A trees and these duplicated genes match with orthologs within α-*Proteobacteria*, a sizeable amount (48) of Type-A genes had homology to bacteria outside of α-*Proteobacteria *(13.33%). These other duplications were found primarily in β-*Proteobacteria *(3.33%) and γ-*Proteobacteria *(7.22%); as shown in Figure 7.

**Table 1:** Distribution of Tree Types and Bootstrap Values in R. sphaeroides

	CI-CI		CI-CII		CII-CII	
**Duplicated Genes**	**116**		**62**		**11**	

	**A-Type**	**B-Type**	**A-Type**	**B-Type**	**A-Type**	**B-Type**

***v *≥ 90**	101	9	47	11	8	3

**70 ≤ *v *< 90**	3	0	0	1	0	0

***v *< 70**	1	2	1	2	0	0

**Total**	105 (91.5%)	11 (9.5%)	48 (77.4%)	14 (22.6%)	8 (72.7%)	3 (27.3%)

Note: Bootstrap Value = *v*

**Figure 8:**
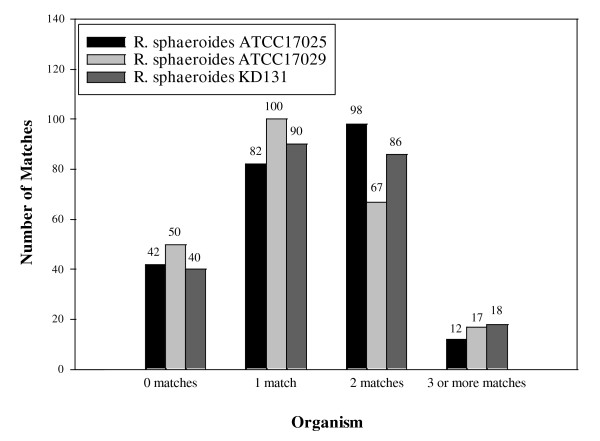
**The distribution of matches to the *R. sphaeroides *2.4.1 query sequences**. BLASTP analysis with each single gene in each of the 234 duplicate gene pairs was performed against three other *R. sphaeroides *strains (ATCC 17025, ATCC 17029, KD131). The matches that met the specified criteria were kept and examined accordingly. The picture depicts varying levels of gene loss and retention among each of the strains.

The number of significant matches (meeting the designated criteria mentioned in the Materials and Method section) of *R. sphaeroides *2.4.1 query protein sequences to three other *R. sphaeroides *strains (ATCC 17025, ATCC 17029, and KD131) was also determined (Additional file 3). The results show that there is significant variability with levels of gene loss and gene retention. Merely 28 (11.97%) of the 234 queries had only two gene matches, representing a duplication pair, in all three strains. 26 (92.86%) of these 28 possessed Type-A gene topology while only 2 (7.14%) possessed Type-B topology. In 144 (61.54%) of the 234 queries, at least one strain had two matches; 122 (84.72%) of the 144 displayed Type-A topology while 22 (15.28%) represented Type-B trees. Figure 8 details the distribution of the matches for the three strains. The match distribution reveals varying levels of gene retention among the organisms. A good deal of genes in the three strains (40 - 50 genes) presented zero matches suggesting that either these genes have been lost from the organisms or they have sufficiently diverged as to not present significant homology to their strain counterparts. In addition, *R. sphaeroides *ATCC 17029 has a much lower number of 2 matches (67) and higher numbers of 0 matches (50) and 1 match (100) in relation to those of the other strains.

**Figure 9:**
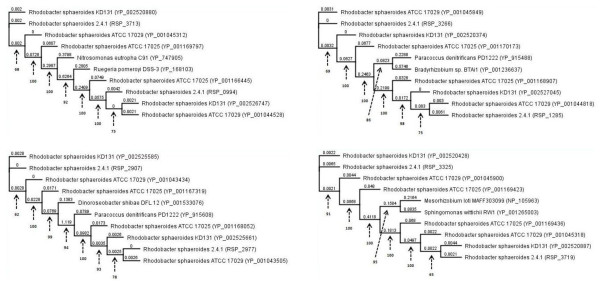
**An expanded tree of four protein pairs**. These maximum likelihood trees include genes from all four *R. sphaeroides *species (2.4.1, ATCC 17025, ATCC 17029, and KD131) along with two related genes from species outside of *R. sphaeroides *(orthologs). These genes in the other *R. sphaeroides *strains were also only present in only two copies. The relationships again depict two types of topology - Type A or Type B, where the left two trees are Type A trees while the right two trees are Type B trees. For the top Type-A tree, the two *R. sphaeroides *2.4.1 genes are *phaD *(RSP_0994) and a hypothetical protein (RSP_3713) while for the bottom Type-A tree the two *R. sphaeroides *2.4.1 genes are *prfA *(RSP_2907) and *prfB *(RSP_2977). For the top Type-B tree, the two *R. sphaeroides *2.4.1 genes are *cbbF1 *(RSP_1285) and *fbpB *(RSP_3266) while for the bottom Type-B tree, both genes encode for hypothetical proteins (RSP_3325 and RSP_3719). The trees were rooted to provide a better sense of the tree topology. The numbers on the branches represent the substitutions per site while the numbers that point to branching points represent the bootstrap support values for those nodes. The NCBI reference number for the corresponding gene is given to the right of the organism description for all nodes except those labeled *R. sphaeroides *2.4.1, where an RSP number is given for consistency with the rest of the information provided in the paper. Notice on the Type A trees how the duplicated genes in all *R. sphaeroides *branch from the orthologs while on the Type B trees the duplications branch from *R. sphaeroides *genes and the orthologs form their own branch.

Figure 9 provides four expanded phylogenetic trees with genes from all four *R. sphaeroides *species (2.4.1, ATCC 17025, ATCC 17029, and KD131) along with two related genes from species outside of *R. sphaeroides *(orthologs). These genes in the other *R. sphaeroides *strains were also only present in only two copies. The relationships again depict two types of topology - Type A or Type B, where two trees are Type A and two trees are Type B. The type A trees were between *phaD *(RSP_0994) and a hypothetical protein (RSP_3713) and between *prfA *(RSP_2907) and *prfB *(RSP_2977). The type B trees were between *cbbF1 *(RSP_1285) and *fbpB *(RSP_3266) and between two hypothetical proteins (RSP_3325 and RSP_3719). The Type A trees demonstrate that one set of genes (the duplicated set) in all *R. sphaeroides *strains branch from the orthologs while on the Type B trees, the duplications branch from *R. sphaeroides *genes while the orthologs form their own branch offshoot. The trees are most probably not instructive in terms of specific strain formation and evolution and so were not treated as such, but rather the genes were viewed in terms two clusters paralleling the two genes in a duplicate pair, where each cluster was a group of directly related *R. sphaeroides *genes.

**Figure 10:**
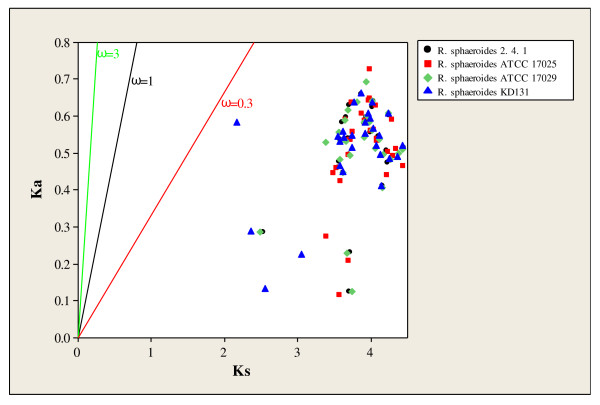
**K_a_-K_s _correlation of 28 common gene pairs in four *R. sphaeroides *strains (2.4.1, ATCC 17025, ATCC 17029, and KD131)**. K_a _and K_s _values were estimated using MYN (Modified Yang-Nielsen algorithm). ω = 0.3, 1, and 3 were used for negative, neutral, and positive selection, respectively.

Analysis on the 28 common gene pairs among the four *R. sphaeroides *strains revealed that the common gene pairs are experiencing similar functional constraints within all four species. The correlation of nonsynonymous (K_a_) and synonymous (K_s_) substitution rates for these gene pairs is shown in Figure 10. Under the modified Yang-Nielsen algorithm, ω = 0.3, 1, and 3 were used for negative, neutral, and positive selection, respectively [37,38]. The correlation data reveals that most of the data points cluster similarly with all ω values less than 0.3, indicative of negative or purifying selection operating on these orthologs. A one-way ANOVA demonstrated that the distributions of ω among the four *R. sphaeroides *strains were not significantly different from one another (*p *= 0.920). For the four strains, the mean ω value varied between 0.131 and 0.137 and the standard deviation of ω varied between 0.030 and 0.037 (pooled S.D. = 0.033).

### Horizontal Gene Transfer

For *R. sphaeroides *2.4.1, the putative HGT regions were found both in CI and CII. The non-optimized coordinates for these regions are not shown. The CI HGT regions sum to 65,005 nucleotides, which spans over 60 genes and which comprises 2.04% of the total CI replicon. The CII HGT regions sum to 110,009 nucleotides, containing 99 genes, and comprises 11.66% of the total CII replicon. Of the 60 HT genes in CI, 5 are among the duplicate gene pairs, while of the 99 HT genes in CII, 8 are among the duplicate gene pairs. The distribution of HGT regions on both chromosomes revealed that most of the duplicated genes are outside of these HGT regions.

## Discussion

### Extent of gene duplication and horizontal gene transfer in *R. sphaeroides*

A systematic genome analysis of the *R. sphaeroides*, which possess multiple chromosomes, has shown approximately the same level of gene duplication (~28%) as reported in many other bacterial genomes that possess only one chromosome [22,42-44] and eukaryotes [22,45-47]. Thus, similar levels of gene duplication in the genomes of eubacteria, archeae, and eukarya suggest that genome size or genome complexity and the levels of gene duplication present in their genomes are not correlated. Gene duplication can occur on two different scales: large-scale duplication (whole-genome duplication, WGD) and smaller-scale duplications, which consists of tandem duplication of short DNA sequence within a gene, duplication of the entire gene or duplication of large genomic segments [48-50].

The majority of gene duplications in *R. sphaeroides *exist in the form of small DNA segments (one or few genes), but a few duplications span over a large segment of genomic segments. For example, chemotaxis-related genes are located at four major loci, chemotaxis operon I (RSP2432-RSP2444), chemotaxis operon II (RSP1582-RSP1589), chemotaxis operon III (RSP0042-RSP0049), and chemotaxis operon IV is a part of a 56 kb- flagella biosynthesis gene cluster (RSP0032-RSP0088). Three copies are present on CI and one copy is present on CII. Although bacteria have acquired a reasonable proportion of their genetic diversity through horizontal gene transfer (HGT) from related microorganisms [17], its percentage varies from 1.5% to 14.47% [16]. The results for *R. sphaeroides *HGT fell within these ranges but the amount of HGT in CII was significantly higher proportionally (11.66%) compared to that in CI (2.04%). Such distinct levels of HGT for CI and CII may suggest that both chromosomes play different roles in *R. sphaeroides*. This observation further confirms that CII has been more flexible in acquiring genes from other species [51]. However, it must be noted that this method of analyzing HGT may not pick up genes that are horizontally transferred between species of similar composition. In addition, although the role of duplicated genes in the majority of bacterial species still remains unclear, the role of gene duplication in the resident genome cannot be underestimated, especially since the majority of these gene duplications are not located within putative HGT regions as seen in *R. sphaeroides*.

### Protein divergence and the evolution of different COG functions in *R. sphaeroides*

Gene duplications in *R. sphaeroides *involved in a wide variety of metabolic functions, and these duplications revealed a considerable variation in amino acid divergence within each metabolic function category. For example, protein pairs involved in flagellar assembly and energy production diverged 60-70%, while protein-pairs involved in photosynthesis and carbon metabolism diverged only 10-30%. These conserved gene homologs may either protect against deleterious changes in either copy and consequently result in functional redundancy or may not have been cleared out simply because they are not harmful to the organism. Two sets of flagellar operons and *neu *operons were located on CI, and most homologous protein pairs had diverged approximately 60-70% of their amino acid sequences. One complete set of flagellar genes (RSP0032-RSP0084) is functional as these genes were expressed in all growth conditions, while the microarray expression of the incomplete flagellar operon (RSP1302-RSP1330) was not detected [52], and therefore the second set of flagellar genes could be required for surface translocation during biofilm production or in an alternative lifestyle that has not been identified yet as seen in other organisms [53,54]. Besides the genes for known functions, the genome of *R. sphaeroides *contains about 40 duplicate genes encoding hypothetical proteins. About one-half of the total hypothetical protein-pairs diverged ~10-20%, and the other half of the hypothetical protein-pairs diverged ~50-70%. The analyses further revealed that genes involved in groups L (DNA synthesis), N (Cell motility and secretion), U (Intracellular transport), C (Energy production), G (Carbohydrate metabolism), and H (Coenzyme metabolism) were overrepresented among genes evolved by gene duplication, while the number of genes representing other COGs remained low or fairly equal percentage-wise to the number of genes representing those COGs in the overall genome of *R. sphaeroides*. Therefore, genes involving transport and metabolism were selected for by gene duplication. In addition, the distribution of the gene duplications (Figure 4) revealed that clusters of gene duplications of the same COG function exist on both CI and CII and that most of the gene duplications in a cluster possessed roughly similar levels of sequence conservation. As such, it may be possible that these highlighted chromosomal segments are locally selected for, especially as these gene duplications possess similar functions.

The sequence similarity and evolutionary constraints of the duplicate gene-pair are indicative of the essential or nonessential nature of gene function. Previous studies have revealed shown that the type II topoisomerases gyrase and topoisomerase IV demonstrated 40 to 60% amino acid sequence identity, but each protein has a distinct function essential for cell survival [55,56] highlighting the limitations in bioinformatics approaches. In a similar note, duplicate protein pairs with very little amino acid identity can share similar functions. In *Bacillus subtilis*, the peptide defomylases (Def and YkrB) show similarity only across short sequences (motifs) but both independently carry a deformylase reaction essential for cell viability [57]. Therefore, gene disruption analysis is further required to determine the definitive function of isologous gene-pairs.

In the specific analysis involving the carbon metabolism genes, it is likely that the cluster in CI containing *cbbA*, *cbbF*, *cbbM*, *cbbP *duplicated first and then *cbbG *and *cbbT *duplications arose from CI and were inserted between the duplicated *cbbA *and *cbbP *genes on CII. In addition, the two genes that code for hypothetical proteins found between *cbbT *and *cbbG *on CI may have arisen through an additional insertion or transposition event. Although these duplicated genes exhibit varying levels of protein divergence, these protein-pairs are under negative selection as evidenced by the functional constraints analysis in Figure 10. Additionally, the identity between the *cbbM *genes was low (31%). This is most probably due to the high degree of difference between *cbbM*_*I *_and *cbbM*_*II*_. More specifically, it has been shown that *cbbM*, which performs the first critical step in carbon fixation, has two forms (*cbbM*_*I *_and *cbbM_II_*). The form I enzymes possesses large and small subunits while the form II enzyme possesses only large subunits that are different from the form I large subunits [58]. The distinguishing between CO_2_/O_2 _is primarily accomplished by loop 6 of the large subunit, which contains a conserved element of 11 amino acid residues. Form II enzymes are primarily anaerobic and unable to function in aerobic environments whereas form I enzymes can function in aerobic environments [59,60]. As form II enzymes are not widely distributed among different species, it is most likely that form I enzymes duplicated to make form II enzymes in certain species and then diverged from its original function to operate in aerobic environments [61]. In contrast, the *cbbA *genes may actually encode for two different enzymes (*cbbA*_*I *_and *cbbA_II_*), although there is high identity between the two genes (79%). *cbbA_II _*genes are usually confined to simple organisms such as bacteria and fungi while *cbbA_I _*is present only some bacteria such as *R. sphaeroides*, but is mostly confined to higher level organisms, including plants and animals. It could be that these two *cbbA *genes in *R. sphaeroides *are therefore different although they share high homology as these two enzymes are thought to have evolved from convergent evolution [62,63]. However, in many instances, there is not markedly homology between *cbbA*_*I *_and *cbbA_II _*[63]. Therefore, the physiological significance of these duplications, including those involving *cbbA *and *cbbM*, need to be further studied biochemically and molecularly to better understand their relationships.

### Ancient gene duplications predated the existence of two chromosomes in *R. sphaeroides*

Since the overwhelming majority of gene duplication in the current day *R. sphaeroides *genome are orthologs and originated prior to or at the time of lineage formation, these findings also validate previous results that a large-scale gene duplication event might have occurred prior to the speciation of *R. sphaeroides *[28]. and possibly even before the diversification of the α-3 *Proteobacteria *[52]. The HGT analysis conducted suggests that the contribution of laterally transferred genes to the duplicated genes is not very significant. It must also be noted that with the sequencing of new organisms and strains, it is possible that new ortholog matches to these gene duplications could be found. However, even so, such new sequences could only change Type-B trees to Type-A trees. Such an understanding aids the mentioned finding that an overwhelming majority of the gene duplications are Type-A. Another issue that must be noted is that it is possible that genes in relatively recent duplications in separate *R. sphaeroides *strains could have evolved to look more like functional homologs in other species. However, 61.54% of the 234 *R. sphaeroides *2.4.1 gene pairs were found in at least one other *R. sphaeroides *strain. Moreover, the functional constraints data among the 28 common gene pairs shows that these pairs are under negative selection and are therefore strongly conserved in function. It is likely then that the majority of gene duplications in *R. sphaeroides *are undergoing negative selection as well.

In addition, the identification of homologous gene pairs among the other three strains of *R. sphaeroides *reveals that although a gene duplication event may have occurred prior to the formation of *R. sphaeroides *lineage, significant gene loss or retention has occurred among all *R. sphaeroides *strains. The distribution of matches on *R. sphaeroides *ATCC 17029 also suggests a greater amount of gene loss or divergence compared to that of the other strains and so this strain may have originated earlier from the lineage compared to the others as it has had more time to undergo selection and deletion processes. However, the genome of *R. sphaeroides *ATCC 17029 revealed high nucleotide identity (~95%) with *R. sphaeroides *2.4.1 in regions of common homology [51], so rather it may be that several duplicate gene pairs have diverged to a level where no protein sequence similarity can be detected.

Since many gene homologues of *R. sphaeroides *share high genetic identity with homologues (orthologs) from a diverse group of α-*Proteobacteria *species, a massive gene duplication event may have had occurred before the diversification of species in α-*Proteobacteria*. The overwhelming presence of Type-A gene duplications on CI and CII unambiguously demonstrates that both chromosomes (CI and CII) were present at the time of species formation, and therefore these two chromosomes have been essential partners within the *R. sphaeroides *genome since its formation.

## Conclusions

The analyses reveal the abundance of gene duplications in *R. sphaeroides *2.4.1 performing a wide range of functions. Moreover, although majority of gene duplications have originated prior to speciation of the *R. sphaeroides *lineage, there are varying amounts of gene loss or conservation among the four *R. sphaeroides *strains. The functional constraints analysis shows that all of the common duplications among the four *R. sphaeroides *strains are under purifying selection suggesting the conservation of the functions of these gene pairs. Finally, the results suggest that the level of gene duplication in organisms with complex genome structuring (more than one chromosome) is not markedly different from that in organisms with only a single chromosome.

## Authors' contributions

All authors (AB, LL, KS, AP, HC, MC) have substantially contributed to the manuscript. All authors were in some part responsible for the collection and analysis of data and formation of the manuscript. All authors approve of the final manuscript.

## Supplementary Material

Additional file 1 **Gene Duplications in *R. sphaeroides *2.4.1**. This file contains detailed information about the distribution and nature of the gene duplications located within *R. sphaeroides *2.4.1. Click here for file

Additional file 2 ***R. sphaeroides *Ortholog Matches**. This file contains detailed information about the highest ortholog matches of each of the proteins in a duplicate pair to bacteria outside of the *R. sphaeroides *species. Click here for file

Additional file 3 ***R. sphaeroides *Strain Hits**. This file contains information concerning the number of hits of a protein in a duplicate pair in *R. sphaeroides *2.4.1 to three other *R. sphaeroides *strains (ATCC 17025, ATCC 17029, and KD131). Click here for file
