# Supplementary material for: The prevalence of gene duplications and their ancient origin in Rhodobacter sphaeroides 2.4.1
Source: BMC Microbiol. 2010 Dec 30;10:331. doi: 10.1186/1471-2180-10-331 (PMC3024229; doi:10.1186/1471-2180-10-331)
Supplement: Additional file 1 — Gene Duplications in R. sphaeroides 2.4.1. This file contains detailed information about the distribution and nature of the gene duplications located within R. sphaeroides 2.4.1. [file 1471-2180-10-331-S1.PDF]

Supplementary Material
Table 1: Gene Duplications in *R. sphaeroides* 2.4.1

NO.	Orf 1[*]	Orf 2[†]	Length[‡]	GENE	E-VALUE	% I[§]	LOCATION[¶]	COG[#] (Pathway[‖])	General Function
1	RSP_0036	RSP_1325	222/139	*flgA/flgA*	2.00E-09	33	CI/CI	2 (NO/NO)	Flagellar Assembly
2	RSP_0047	RSP_1588	365/369	*cheB2/cheB1*	2.00E-59	42	CI/CI	2 (NT/NT)	Bacterial Chemotaxis
3	RSP_0054	RSP_2220	340/357	*fliG/fliG*	4.00E-12	24	CI/CI	2 (N/N)	Flagellar Assembly
4	RSP_0056	RSP_1332	442/436	*fliI/-*	2.00E-63	39	CI/CI	2 (NU/NU)	Flagellar Assembly
5	RSP_0061	RSP_1310	98/88	*fliN/-*	3.00E-09	35	CI/CI	2 (NU/NU)	Flagellar Assembly
6	RSP_0064	RSP_1328	88/88	*fliQ/fliQ*	9.00E-16	45	CI/CI	2 (N/N)	Flagellar Assembly
7	RSP_0065	RSP_1321	269/260	*fliR/fliR*	3.00E-17	34	CI/CI	2 (NU/NU)	Flagellar Assembly
8	RSP_0074	RSP_1304	1363/481	*flgK1/flgK*	3.00E-09	29	CI/CI	2 (N/N)	Flagellar Assembly
9	RSP_0077	RSP_1324	222/239	*flgH/flgH*	9.00E-19	29	CI/CI	2 (N/N)	Flagellar Assembly
10	RSP_0079	RSP_1327	251/239	*flgF/flgF*	1.00E-16	29	CI/CI	2 (N/N)	Flagellar Assembly
11	RSP_0082	RSP_1330	138/128	*flgC/flgC*	6.00E-09	28	CI/CI	2 (N/N)	Flagellar Assembly
12	RSP_0083	RSP_1331	128/127	*flgB/flgB*	1.00E-08	31	CI/CI	2 (N/N)	Flagellar Assembly
13	RSP_0100	RSP_2512	128/121	*nuoA/nuoA*	1.00E-14	36	CI/CI	3 (C/C)	Energy Production And Conversion
14	RSP_0102	RSP_2515	580/404	*nuoCD/nuoD*	2.00E-90	41	CI/CI	3 (C/C)	Energy Production And Conversion
15	RSP_0104	RSP_2518	416/431	*nuoF/nuoF*	4.00E-77	38	CI/CI	3 (C/C)	Energy Production And Conversion
16	RSP_0105	RSP_2521	860/654	*nuoG/nuoG*	1.00E-51	28	CI/CI	3 (C/C)	Energy Production And Conversion
17	RSP_0108	RSP_2525	164/204	*nuoJ/nuoJ*	1.00E-17	31	CI/CI	0/3 (-/C)	Energy Production And Conversion
18	RSP_0112	RSP_2530	447/478	*nuoN/nuoN*	1.00E-38	32	CI/CI	3 (C/C)	Energy Production And Conversion
19	RSP_0146	RSP_0889	112/112	*glnB/glnK*	4.00E-42	62	CI/CI	3 (E/E)	Energy Production And Conversion
20	RSP_0153	RSP_2225	225/223	-/-	1.00E-41	43	CI/CI	0 (-/-)	Hypothetical Protein
21	RSP_0161	RSP_1883	366/363	-/-	5.00E-150	69	CI/CI	3 (E/E)	Abc Type Transporter
22	RSP_0176	RSP_3074	593/612	*-/ilvD*	2.00E-50	28	CI/CII	3 (EG/EG)	Amino Acid Metabolism
23	RSP_0180	RSP_3166	283/281	-/-	2.00E-27	30	CI/CII	3 (P/P)	Abc Type Transporter
24	RSP_0183	RSP_3462	501/485	*dht/-*	4.00E-78	36	CI/CII	3 (F/F)	Pyrimidine Metabolism
25	RSP_0189	RSP_1149	445/475	*-/gltD*	1.00E-39	31	CI/CI	3/4 (ER/ER)	Nitrogen Metabolism
26	RSP_0224	RSP_0658	723/799	-/-	6.00E-39	31	CI/CI	4 (R/R)	Folate And Starch Metabolism
27	RSP_0229	RSP_2238	532/507	-/-	5.00E-52	31	CI/CI	3 (G/G)	Multidrug Efflux Transposter
28	RSP_0235	RSP_3050	344/338	*moaA/moaA*	2.00E-123	65	CI/CII	3 (H/H)	Molybdenum Biosynthesis
29	RSP_0248	RSP_0479	187/188	-/-	1.00E-40	36	CI/CI	1 (J/J)	Translation (Elongation Factor P)
30	RSP_0254	RSP_1134	648/637	*dxsA/Dxs*	0.00E+00	66	CI/CI	3 (HI/HI)	Isoprenoid Synthesis
31	RSP_0287	RSP_2827	1193/1078	*bchH/cobN*	1.00E-68	27	CI/CI	3 (H/H)	Porphyrin And Chlorophyll Metabolism
32	RSP_0314	RSP_1556	51/51	*puc1B/puc2B*	3.00E-27	94	CI/CI	0 (-/-)	Light Entrapment
33	RSP_0329	RSP_3513	336/326	-/-	2.00E-74	43	CI/CII	2 (T/T)	Signal Transduction System
34	RSP_0345	RSP_3560	334/364	-/-	3.00E-32	30	CI/CII	4 (R/R)	Abc Type Transporter
35	RSP_0382	RSP_1257	601/573	*-/phbC*	4.00E-106	36	CI/CI	3 (I/I)	Butanoate Metabolism
36	RSP_0413	RSP_4018	461/311	-/-	3.00E-27	40	CI/PB	2 (T/T)	Signal Transduction System
37	RSP_0423	RSP_3201	346/339	-/-	1.00E-33	33	CI/CII	3 (C/C)	Energy Production And Conversion
38	RSP_0476	RSP_2364	214/218	*/fucA*	6.00E-93	71	CI/CI	3 (G/G)	Signal Metabolism
39	RSP_0480	RSP_0096	455/477	*uxuB/mtlK*	5.00E-84	41	CI/CI	3 (G/G)	Carbohydrate Metabolism
40	RSP_0563	RSP_2815	306/311	*xerC/xerD*	1.00E-34	35	CI/CI	1 (L/L)	Site-Specific Integrase And Recombinase
41	RSP_0576	RSP_3142	605/576	*-/actP*	2.00E-29	30	CI/CII	4 (R/R)	Na+/Solute Symporter
42	RSP_0578	RSP_1736	217/217	*-/adk*	3.00E-57	46	CI/CI	3 (F/F)	Purine Metabolism
43	RSP_0601	RSP_2410	292/298	*rpoH2/rpoH1*	1.00E-62	47	CI/CI	1 (K/K)	Transcription (Sigma-32)
44	RSP_0632	RSP_2155	434/441	*hisD/hisD*	4.00E-60	38	CI/CI	3 (E/E)	Amino Acid Metabolism
45	RSP_0656	RSP_3330	592/592	*-/sac1*	0.00E+00	64	CI/CII	3 (P/P)	Sodium/Sulphate Symporter
46	RSP_0672	RSP_0920	232/349	*tolQ/exbB*	4.00E-31	40	CI/CI	2 (U/U)	Transporter
47	RSP_0688	RSP_0550	501/355	-/-	5.00E-37	32	CI/CI	2 (M/M)	Penicillin-Binding Protein
48	RSP_0692	RSP_3027	477/469	*rdxB/rdxA*	0.00E+00	67	CI/CII	3 (C/C)	Energy Production And Conversion
49	RSP_0698	RSP_2572	248/263	*fnrL/crpK*	1.00E-35	35	CI/CI	2 (T/T)	Crp-Fnr Regulator
50	RSP_0723	RSP_0240	372/523	*ald/pntA*	1.00E-36	31	CI/CI	3 (E/C)	Carbon Fixation
51	RSP_0759	RSP_4086	378/360	-/-	7.00E-26	29	CI/PC	2 (M/M)	Transporter
52	RSP_0766	RSP_0013	336/294	-/-	2.00E-70	49	CI/CI	4 (S/S)	Hypothetical Protein
53	RSP_0772	RSP_1346	663/811	*parE/gyrB*	2.00E-116	43	CI/CI	1 (L/L)	Dna Replication
54	RSP_0817	RSP_2004	390/503	*-/trpE*	2.00E-39	39	CI/CI	3 (EH/EH)	Amino Acid Metabolism
55	RSP_0840	RSP_1989	847/ 987	*leuS/valS*	1.00E-25	25	CI/CI	1 (J/J)	Translation (Trna Synthetase)
56	RSP_0893	RSP_1120	433/433	-/-	3.00E-32	28	CI/CI	2 (V/V)	Type 1 Secretion System
57	RSP_0902	RSP_0089	214/228	-/-	4.00E-30	39	CI/CI	4 (R/R)	Hydrolase
58	RSP_0945	RSP_3035	541/425	-/-	5.00E-29	30	CI/CII	0 (-/-)	Transporter
59	RSP_0953	RSP_2415	794/463	-/-	2.00E-68	30	CI/CI	4 (S/S)	Hypothetical Protein
60	RSP_0961	RSP_2192	652/709	*meaA/mcmA*	1.00E-111	38	CI/CI	3 (I/I)	Amino Acid Metabolism
61	RSP_0970	RSP_1771	285/318	-/-	5.00E-42	35	CI/CI	3 (G/G)	Tca Cycle
62	RSP_0971	RSP_2392	184/185	-/-	1.00E-55	52	CI/CI	4 (S/S)	Hypothetical Protein
63	RSP_0976	RSP_3147	600/587	*sdhA/sdhA*	5.00E-62	32	CI/CII	3 (C/C)	Butanoate Metabolism And Tca Cycle
64	RSP_0979	RSP_3150	259/242	*sdhB/frdB*	2.00E-32	35	CI/CII	3 (C/C)	Butanoate Metabolism And Tca Cycle
65	RSP_0990	RSP_3687	340/310	-/-	2.00E-24	28	CI/CII	3 (G/G)	Abc Type Transporter
66	RSP_0992	RSP_3715	949/964	*phaA/B/*	0.00E+00	45	CI/CII	3 (CP/CP)	Monovalent Cation/H+ Antiporter
67	RSP_0993	RSP_3714	111/114	*phaC/-*	2.00E-29	50	CI/CII	3 (P/P)	Monovalent Cation/H+ Antiporter
68	RSP_0994	RSP_3713	503/540	*phaD/-*	2.00E-79	41	CI/CII	3 (CP/CP)	Monovalent Antitransporter
69	RSP_1013	RSP_2814	424/442	*-/corB*	1.00E-36	28	CI/CI	3/4 (R/P)	Hemolysin-Like Protein
70	RSP_1016	RSP_1572	158/155	-/-	5.00E-37	50	CI/CI	2 (O/O)	Small Heat Shock Protein
71	RSP_1036	RSP_1035	180/187	*atpX/atpF*	2.00E-04	30	CI/CI	3/0 (C/-)	Atp Synthesis

72	RSP_1097	RSP_1832	448/419	-/-	1.00E-31	30	CI/CI	4 (R/R)	Zinc Protease And Peptidase
73	RSP_1109	RSP_2147	358/344	*cysK/cysK*	6.00E-40	36	CI/CI	3 (E/E)	Amino Acid Metabolism
74	RSP_1123	RSP_3553	940/1163	*dnaE/dnaE*	4.00E-92	30	CI/CII	1 (L/L)	Nucleic Acid Metabolism
75	RSP_1184	RSP_2660	278/373	-/-	4.00E-25	33	CI/CI	4 (S/S)	Ribonuclease
76	RSP_1255	RSP_2305	473/147	-/-	1.00E-34	51	CI/CI	3 (C/I)	Bifunctional Enoyl-Coa Hydratase/MaoC
77	RSP_1260	RSP_6198	153/148	-/-	2.00E-43	53	CI/CII	0 (-/-)	Hypothetical Protein
78	RSP_1261	RSP_4060	140/134	*blrB/blrA*	6.00E-47	61	CI/PC	0 (-/-)	Blue-Light Receptor
79	RSP_1272	RSP_2681	194/168	*-/rpoE*	3.00E-38	48	CI/CI	1 (K/K)	Transcription (Sigma-70)
80	RSP_1278	RSP_2504	218/229	*cbbZ/gph*	2.00E-32	39	CI/CI	4 (R/R)	Glycoxylate Metabolism
81	RSP_1282	RSP_3271	486/459	*rbcL/rbpL*	1.00E-39	31	CI/CII	3 (G/G)	Carbon Fixation
82	RSP_1283	RSP_3270	359/354	*cfxA/cfxB*	2.00E-175	79	CI/CII	3 (G/G)	Carbon Metabolism
83	RSP_1284	RSP_3267	290/292	*prkA/prkB*	5.00E-157	87	CI/CII	3 (C/C)	Carbon Metabolism
84	RSP_1285	RSP_3266	333/331	*cbbF1/fbpB*	6.00E-128	68	CI/CII	3 (G/G)	Carbon Metabolism
85	RSP_1303	RSP_0080	465/423	*flgE/flgE*	3.00E-28	25	CI/CI	2 (N/N)	Flagellar Assembly
86	RSP_1307	RSP_0076	363/371	*flgI/flgI*	1.00E-70	43	CI/CI	2 (N/N)	Flagellar Assembly
87	RSP_1309	RSP_0063	239/301	*fliP/fliP*	5.00E-49	40	CI/CI	2 (NU/NU)	Flagellar Assembly
88	RSP_1312	RSP_0053	509/570	*fliF/fliF1*	2.00E-27	30	CI/CI	2 (NU/NU)	Flagellar Assembly
89	RSP_1319	RSP_0072	226/265	-/-	2.00E-31	45	CI/CI	0 (-/-)	Invasion Protein
90	RSP_1320	RSP_0034	688/704	*flhA/flhA*	6.00E-105	36	CI/CI	2 (NU/NU)	Flagellar Assembly
91	RSP_1322	RSP_0066	359/380	*fhlB/flhB*	8.00E-37	33	CI/CI	2 (NU/NU)	Flagellar Assembly
92	RSP_1326	RSP_0078	261/262	*flgG/flgG*	2.00E-58	42	CI/CI	2 (N/N)	Flagellar Assembly
93	RSP_1379	RSP_2899	479/489	-/-	5.00E-52	38	CI/CI	3 (E/E)	Peptidase
94	RSP_1463	RSP_3181	381/393	-/-	6.00E-33	30	CI/CII	3 (C/C)	Energy Production And Conversion
95	RSP_1492	RSP_1869	409/399	-/-	4.00E-51	35	CI/CI	3 (HC/HC)	Energy Production And Conversion
96	RSP_1499	RSP_3272	525/595	*-/ggt*	3.00E-53	29	CI/CII	3 (E/E)	Amino Acid Transport And Metabolism
97	RSP_1513	RSP_3585	401/409	*trpB/-*	9.00E-100	45	CI/CII	3 (E/E)	Amino Acid Metabolism
98	RSP_1532	RSP_0196	433/421	*hslU/clpX*	7.00E-26	46	CI/CI	2 (O/O)	Protease
99	RSP_1551	RSP_0608	126/127	-/-	4.00E-22	42	CI/CI	4 (R/R)	Glyoxalase Family Protein
100	RSP_1574	RSP_0820	158/169	-/-	1.00E-21	35	CI/CI	3 (C/C)	Energy Oxidation
101	RSP_1591	RSP_3122	333/324	-/-	9.00E-91	53	CI/CII	2 (O/O)	Glutathione S-Transferase
102	RSP_1613	RSP_1413	317/324	-/-	1.00E-42	32	CI/CII	4 (R/R)	Transport
103	RSP_1614	RSP_1412	705/877	-/-	5.00E-80	33	CI/CII	4 (R/R)	Transport
104	RSP_1638	RSP_2062	124/128	-/-	1.00E-56	95	CI/CI	0 (-/-)	Hypothetical Protein
105	RSP_1645	RSP_3652	444/393	-/-	2.00E-138	96	CI/CII	4/0 (R/-)	Phage-Related Protein
106	RSP_1647	RSP_3650	319/305	-/-	3.00E-141	87	CI/CII	2 (OU/OU)	Serine Proteases
107	RSP_1650	RSP_2352	552/510	-/-	2.00E-56	32	CI/CI	4 (R/R)	Phage Terminase-Like Protein
108	RSP_1653	RSP_6190	209/206	-/-	4.00E-101	82	CI/CII	0 (-/-)	Hypothetical Protein
109	RSP_1662	RSP_2080	348/341	-/-	4.00E-65	39	CI/CI	1 (L/L)	Integrase-Recombinase
110	RSP_1696	RSP_2630	767/907	*-/gyrA*	2.00E-122	39	CI/CI	1 (L/L)	Dna Replication
111	RSP_1706	RSP_1121	214/268	-/-	1.00E-24	40	CI/CI	4 (R/R)	Xenobiotic Acyltransferase
112	RSP_1766	RSP_1848	481/508	*pykA/-*	7.00E-36	35	CI/CI	3 (G/G)	Glycolysis
113	RSP_1767	RSP_2323	245/254	-/-	9.00E-31	37	CI/CI	3 (E/E)	Hypothetical Protein
114	RSP_1787	RSP_2334	328/314	*fruK/pfkB*	1.00E-28	37	CI/CI	3 (G/G)	Carbon Metabolism
115	RSP_1820	RSP_0088	169/166	-/-	2.00E-39	53	CI/CI	4 (S/S)	Hypothetical Protein
116	RSP_1843	RSP_1053	393/505	*ftsY/ffh*	9.00E-39	33	CI/CI	2 (U/U)	Protein Export
117	RSP_1850	RSP_1786	747/838	*ptsI/fruB*	2.00E-50	35	CI/CI	2/3 (T/G)	Carbohydrate Metabolism
118	RSP_1889	RSP_3093	246/237	-/-	1.00E-57	49	CI/CII	4 (R/R)	Hypothetical Protein
119	RSP_1894	RSP_2496	129/166	-/-	1.00E-64	81	CI/CI	2 (M/M)	Hypothetical Protein
120	RSP_1927	RSP_3172	272/277	-/-	4.00E-41	39	CI/CII	4 (R/R)	Hypothetical Protein
121	RSP_1931	RSP_2653	304/279	-/-	3.00E-32	31	CI/CI	4 (R/R)	Gas Vesicle Protein
122	RSP_1944	RSP_2820	254/241	*-/cobA*	7.00E-30	42	CI/CI	3 (H/H)	Porphyrin And Chlorophyll Metabolism
123	RSP_1951	RSP_3622	116/116	-/-	3.00E-65	96	CI/CII	0 (-/-)	Hypothetical Protein
124	RSP_1954	RSP_6191	88/79	-/-	4.00E-22	60	CI/CII	0 (-/-)	Hypothetical Protein
125	RSP_1955	RSP_3647	262/175	-/-	4.00E-52	72	CI/CII	0 (-/-)	Hypothetical Protein
126	RSP_1956	RSP_6196	124/124	-/-	7.00E-66	92	CI/CII	0 (-/-)	Hypothetical Protein
127	RSP_1966	RSP_3007	291/193	-/-	7.00E-75	99	CI/CII	1/0 (L/-)	Transposase
128	RSP_1984	RSP_2284	381/361	-/-	3.00E-31	30	CI/CI	3 (E/E)	Amino Acid Metabolism
129	RSP_1998	RSP_3049	394/410	*-/moeA*	1.00E-50	38	CI/CII	3 (H/H)	Molybdopterin Biosynthesis
130	RSP_2061	RSP_1637	61/78	-/-	2.00E-24	85	CI/CI	0 (-/-)	Hypothetical Protein
131	RSP_2063	RSP_1639	147/146	-/-	6.00E-79	91	CI/CI	0 (-/-)	Hypothetical Protein
132	RSP_2064	RSP_6012	126/126	-/-	5.00E-66	89	CI/CI	0 (-/-)	Hypothetical Protein
133	RSP_2065	RSP_1640	185/157	-/-	8.00E-28	39	CI/CI	0 (-/-)	Hypothetical Protein
134	RSP_2098	RSP_2326	597/648	*ftsI/pbpA*	2.00E-26	27	CI/CI	2 (M/M)	Cell Division
135	RSP_2106	RSP_2325	388/379	*ftsW/rodA*	3.00E-30	31	CI/CI	2 (D/D)	Cell Division
136	RSP_2122	RSP_3346	232/908	*mtbC/-*	3.00E-27	33	CI/CII	3/4 (R/E)	Amino Acid Transport And Metabolism
137	RSP_2124	RSP_3347	335/359	-/-	7.00E-29	32	CI/CII	3 (E/E)	Amino Acid Metabolism
138	RSP_2184	RSP_3292	548/533	*betA/-*	3.00E-100	39	CI/CII	3 (E/E)	Glucose-Methanol-Choline Oxidoreductase
139	RSP_2189	RSP_2508	510/558	*pccB/mccB*	4.00E-64	32	CI/CI	3 (I/I)	Amino Acid Metabolism
140	RSP_2201	RSP_2200	126/132	-/-	6.00E-28	50	CI/CI	1 (K/K)	Transcriptional Regulator
141	RSP_2227	RSP_2886	409/423	*glgC/glgC*	5.00E-67	38	CI/CI	3 (G/G)	Glucose Metabolism
142	RSP_2232	RSP_1001	474/260	-/-	2.00E-23	39	CI/CI	1 (L/L)	Dna Repair
143	RSP_2247	RSP_1708	669/705	*fusA/fusA1*	6.00E-77	28	CI/CI	1 (J/J)	Translation (Elongation Factor G)
144	RSP_2297	RSP_3935	512/497	*atpA/atpA*	6.00E-119	46	CI/PA	3 (C/C)	Atp Synthesis
145	RSP_2360	RSP_2997	397/408	-/-	2.00E-28	29	CI/CI	4 (S/S)	Phage-Related Protein
146	RSP_2397	RSP_3040	339/385	-/-	5.00E-37	32	CI/CII	3 (E/E)	Abc Type Transporter

147	RSP_2441	RSP_1589	595/580	*tlpS/tlpC*	8.00E-31	26	CI/CI	0 (-/-)	Bacterial Chemotaxis
148	RSP_2459	RSP_2809	260/366	-/-	3.00E-30	34	CI/CI	3 (Q/Q)	Abc Type Transporter
149	RSP_2470	RSP_2996	187/174	-/-	9.00E-36	51	CI/CI	4 (R/R)	Peptidase
150	RSP_2482	RSP_4189	294/294	*TonB/TonB*	9.00E-163	94	PC/PD	1 (L/L)	Tonb-Dependent Receptor Protein
151	RSP_2501	RSP_3873	279/277	*-/modD*	1.00E-37	46	CI/PA	3 (H/H)	Molybdenum Transport
152	RSP_2513	RSP_0101	174/205	*nuoB/nuoB*	2.00E-41	53	CI/CI	3 (C/C)	Energy Production And Conversion
153	RSP_2522	RSP_0106	345/318	*nuoH/nuoH*	5.00E-61	43	CI/CI	3 (C/C)	Energy Production And Conversion
154	RSP_2523	RSP_0107	167/164	*nuoI/nuoI*	1.00E-29	43	CI/CI	3 (C/C)	Energy Production And Conversion
155	RSP_2527	RSP_0110	72/700	*nuoL/nuoL*	2.00E-57	34	CI/CI	3 (CP/CP)	Energy Production And Conversion
156	RSP_2565	RSP_1159	312/297	*ugpG/galU*	2.00E-119	66	CI/CI	2 (M/M)	Galactose Metabolism
157	RSP_2592	RSP_2881	386/390	*-/gfdT*	2.00E-72	43	CI/CI	4 (S/S)	Hypothetical Protein
158	RSP_2607	RSP_3017	166/172	-/-	3.00E-33	42	CI/CII	4 (R/R)	Amino Acid Metabolism
159	RSP_2618	RSP_3826	250/262	-/-	2.00E-31	35	CI/CII	4 (R/R)	Hemolysin-Like Protein
160	RSP_2623	RSP_2850	695/1165	*recG/mfd*	3.00E-61	35	CI/CI	1 (LK/LK)	Dna Repair System
161	RSP_2673	RSP_2578	781/600	*/xoxF*	4.00E-36	25	CI/CI	3 (G/G)	Pentose Phosphate Pathway
162	RSP_2749	RSP_0217	439/413	*-/int*	4.00E-23	27	CI/CI	1 (L/L)	Phage-Related Integrase
163	RSP_2779	RSP_2380	488/710	*catA/catC*	1.00E-100	42	CI/CI	3 (P/P)	Detoxification Of Cells
164	RSP_2781	RSP_0899	182/162	-/-	1.00E-25	36	CI/CI	2 (O/O)	Thiol Peroxidase
165	RSP_2846	RSP_0154	296/290	-/-	1.00E-28	36	CI/CI	3 (I/I)	3-Hydroxyisobutyrate Dehydrogenase
166	RSP_2856	RSP_0704	603/645	-/-	2.00E-104	39	CI/CI	3 (E/E)	Abc Type Transporter
167	RSP_2888	RSP_3341	144/145	-/-	2.00E-27	38	CI/CII	1 (K/K)	Transcriptional Regulator
168	RSP_2907	RSP_2977	351/375	*prfA/prfB*	3.00E-58	42	CI/CI	1 (J/J)	Translation (Peptide Chain Release Factor)
169	RSP_2909	RSP_3743	321/320	*-/speB1*	1.00E-53	40	CI/CII	3 (E/E)	Amino Acid Metabolism
170	RSP_3015	RSP_3969	306/310	-/-	3.00E-103	56	CII/PB	4 (S/S)	Hypothetical Protein
171	RSP_3028	RSP_2984	407/407	*hemT/hemA*	4.00E-127	54	CII/CI	3 (H/H)	Tetrapyrrole Synthesis
172	RSP_3046	RSP_4118	392/227	*dorC/napC*	7.00E-52	48	CII/PC	3 (C/C)	Energy Production And Conversion
173	RSP_3098	RSP_0118	261/324	*qoxB/-*	2.00E-28	33	CII/CI	3 (C/C)	Energy Production And Conversion
174	RSP_3106	RSP_4276	274/325	*-/zitB*	8.00E-24	32	CII/PE	3 (P/P)	Cation Efflux Protein
175	RSP_3113	RSP_2316	436/413	*dadA/dadA*	6.00E-26	28	CII/CI	3 (E/E)	Nitrogen Metabolism
176	RSP_3116	RSP_3345	388/432	-/-	3.00E-29	31	CII/CII	4 (R/R)	Transporter
177	RSP_3134	RSP_3475	427/668	-/-	1.00E-39	34	CII/CII	1/2/4 (RTKL/RTKL)	Two Component Signal Transduction
178	RSP_3144	RSP_4010	332/339	-/-	2.00E-70	52	CII/PB	4 (S/S)	Hypothetical Protein
179	RSP_3187	RSP_0125	531/540	*mdoD/mdoG*	3.00E-99	43	CII/CI	3 (P/P)	Glucan Biosynthesis
180	RSP_3253	RSP_3443	319/321	-/-	2.00E-36	38	CII/CII	3 (C/C)	Acetamidase/Formamidase
181	RSP_3268	RSP_2956	657/672	*tklB/tktA*	0.00E+00	58	CII/CI	3 (G/G)	Carbon Metabolism
182	RSP_3275	RSP_1024	323/338	-/-	9.00E-59	45	CII/CI	4 (R/R)	Moxr-Like Protein
183	RSP_3288	RSP_1444	287/282	*ugpE/-*	3.00E-31	31	CII/CI	3 (G/G)	Abc Type Transporter
184	RSP_3325	RSP_3719	501/504	-/-	5.00E-144	58	CII/CII	4 (R/R)	Polysaccharide Biosynthesis
185	RSP_3342	RSP_1546	165/161	*bfr/bfr*	9.00E-81	84	CII/CI	3 (P/P)	Bacterial Bacterioferritin
186	RSP_3349	RSP_4072	292/295	-/-	1.00E-99	64	CII/PC	0 (-/-)	Hypothetical Protein
187	RSP_3350	RSP_4071	317/362	-/-	9.00E-122	64	CII/PC	0 (-/-)	Hypothetical Protein
188	RSP_3372	RSP_0097	379/365	*-/somM*	1.00E-43	32	CII/CI	3 (Q/Q)	Trap-T Family Transporter
189	RSP_3397	RSP_3515	347/345	-/-	2.00E-29	29	CII/CII	3 (E/E)	Abc Type Transporter
190	RSP_3398	RSP_3657	217/218	-/-	3.00E-30	37	CII/CII	3 (H/H)	Hypothetical Protein
191	RSP_3406	RSP_2904	328/322	*pdxA/pdxA*	4.00E-55	39	CII/CI	3 (H/H)	Vitamin B6 Metabolism
192	RSP_3410	RSP_3386	317/318	-/-	2.00E-27	30	CII/CII	4 (S/S)	Trap-T Family Transporter
193	RSP_3436	RSP_6165	293/298	-/-	2.00E-41	37	CII/CII	1 (K/K)	Lys R Family Transcriptional Regulator
194	RSP_3497	RSP_4069	971/482	-/-	2.00E-29	31	CII/PC	4 (R/R)	Caspase-1, P20
195	RSP_3505	RSP_0090	312/317	*-/smoC*	6.00E-31	31	CII/CI	1 (K/K)	Deor Family Transcriptional Regulator
196	RSP_3547	RSP_2495	755/1218	*-/nrd*	6.00E-31	31	CII/CI	3 (F/F)	Nucleic Acid Metabolism
197	RSP_3574	RSP_2935	523/507	*hutH/hutH*	4.00E-64	36	CII/CI	3 (E/E)	Amino Acid Metabolism
198	RSP_3608	RSP_4107	539/564	-/-	4.00E-59	32	CII/PC	1 (L/L)	Site-Specific Recombinase
199	RSP_3624	RSP_3792	238/238	-/-	5.00E-126	92	CII/CII	0 (-/-)	Hypothetical Protein
200	RSP_3627	RSP_3784	448/448	-/-	0.00E+00	95	CII/CII	2 (OU/OU)	Serine Peptidase
201	RSP_3628	RSP_3786	103/108	-/-	3.00E-46	84	CII/CII	0 (-/-)	Hypothetical Protein
202	RSP_3664	RSP_3405	249/239	*-/gntR*	8.00E-28	34	CII/CII	1 (K/K)	Gntr Family Transcriptional Regulator
203	RSP_3695	RSP_0237	364/387	*argE/argE*	4.00E-26	31	CII/CI	3 (E/E)	Acetylornithine Deacetylase
204	RSP_3697	RSP_3861	330/334	*cysP/-*	2.00E-80	47	CII/PA	3 (P/P)	Abc Type Transporter
205	RSP_3703	RSP_1179	415/432	*-/xylH*	7.00E-38	31	CII/CI	3 (G/G)	Abc Type Transporter
206	RSP_3843	RSP_0024	187/177	*rfbC/rfbC*	2.00E-36	39	PA/CI	2 (M/M)	Dtdp-4-Dehydrorhamnose 3,5-Epimerase
207	RSP_3884	RSP_3887	391/393	-/-	2.00E-26	31	PA/PA	2/4 (MR/MR)	Cell Envelope Biogenesis
208	RSP_3894	RSP_3978	141/410	-/-	1.00E-72	96	PA/PB	3/4 (PR/PR)	Inorganic Transport And Metabolism
209	RSP_3904	RSP_7352	1111/164	*traI/-*	6.00E-36	98	PA/PE	1/0 (L/-)	Congugation Transfer
210	RSP_3906	RSP_2981	273/247	-/-	2.00E-35	35	PA/CI	2 (O/O)	Oxidoreductase/Disulfide Isomerase
211	RSP_3908	RSP_4251	185/185	-/-	2.00E-107	99	PA/PD	0 (-/-)	Hypothetical Protein
212	RSP_3955	RSP_0726	499/471	-/-	3.00E-63	36	PB/CI	3 (E/E)	Amino Acid Metabolism
213	RSP_3956	RSP_3940	337/312	-/-	5.00E-41	35	PB/PB	2 (M/M)	Sugar Isomerase
214	RSP_3985	RSP_3920	488/452	-/-	6.00E-97	46	PB/PA	4 (S/S)	Hypothetical Protein
215	RSP_4008	RSP_2813	517/513	*-/mttB*	3.00E-126	42	PB/CI	3 (H/H)	Trimethylamine Methyltransferase
216	RSP_4021	RSP_7387	433/403	*repC2/-*	5.00E-68	39	PB/PD	0 (-/-)	Dna Replication
217	RSP_4022	RSP_4185	316/328	*repB1/repB*	4.00E-40	35	PB/PD	1 (K/K)	Replication
218	RSP_4050	RSP_4049	442/463	*pdhB/pdhAb*	9.00E-42	66	CI/CI	3 (C/C)	Branched-Chain Acid Dehydrogenase
219	RSP_4053	RSP_0834	475/484	*xanB/xanB*	3.00E-173	67	PC/CI	2/3 (M/G)	Sugar Metabolism
220	RSP_4103	RSP_1428	473/473	-/-	6.00E-59	41	PC/CI	0 (-/-)	Hypothetical Protein
221	RSP_4138	RSP_3902	191/339	-/-	4.00E-110	96	PD/PA	0 (-/-)	Hypothetical Protein

222	RSP_4139	RSP_3901	241/298	*parA/virc1*	7.00E-87	61	PD/PA	2 (D/D)	Cell Division
223	RSP_4165	RSP_4182	523/333	-/-	5.00E-77	43	PD/PD	1 (L/L)	Transposase (Is66)
224	RSP_4178	RSP_3012	102/136	-/-	3.00E-40	74	PD/CII	0/1 (-/L)	Transposase (Isrm14)
225	RSP_4207	RSP_3721	375/383	-/-	4.00E-142	66	PD/CII	2 (M/M)	Glycosyl Transferase/Hypothetical Protein
226	RSP_4209	RSP_3723	392/384	-/-	4.00E-131	71	PD/CII	3 (I/I)	Acyltransferase
227	RSP_4252	RSP_3907	163/163	-/-	3.00E-93	98	PD/PA	0 (-/-)	Hypothetical Protein
228	RSP_6015	RSP_6036	80/76	-/-	3.00E-27	65	CI/CI	0 (-/-)	Hypothetical Protein
229	RSP_6035	RSP_3772	112/113	-/-	3.00E-39	64	CI/CII	0 (-/-)	Hypothetical Protein
230	RSP_6194	RSP_6200	139/140	-/-	8.00E-68	96	CII/CII	0 (-/-)	Hypothetical Protein
231	RSP_6234	RSP_6250	338/310	-/-	1.00E-57	39	CII/CI	0 (-/-)	Hypothetical Protein
232	RSP_6256	RSP_6158	54/263	*puc1A/puc2A*	3.00E-16	58	CI/CI	0 (-/-)	Light Entrapment
233	RSP_7246	RSP_4028	354/354	-/-	1.00E-129	67	PC/PB	3 (I/I)	Acyltransferase
234	RSP_7390	RSP_3896	405/420	*-/repC*	0.00E+00	85	PD/PA	0 (-/-)	Transposase

[*] The genes used to search against the genome of *R. sphaeroides* 2. 4. 1 for the homologs

[†] The genes sharing the highest identity with the gene used to search against the genome

[‡] The polypeptide lengths of the protein pairs (Orf1 and Orf2)

[§] Percent Amino Acid Identity between the two protein pairs (Orf1 and Orf2)

[¶] Location (Orf1 location/Orf2 location) of the gene duplications: CI – Chromosome I; CII – Chromosome II; PA – Plasmid A; PB – Plasmid B; PC – Plasmid C; PD – Plasmid D; PE – Plasmid E

[#] Clusters of Orthologous Groups of proteins (COGs): 0. Not in COGs (-); 1. Information storage and processing (J/ A/ K/ L/ B); 2. Cellular processes (D/V/T/M/N/Z/U/O); 3. Metabolism (C/ G/ E/ F/ H/ I/ P/ Q); 4. Poorly characterized (R/S)

[||] J. Translation, ribosomal structure and biogenesis; A. RNA processing and modification; K. Transcription; L. DNA replication, recombination and repair; B. Chromatin structure and dynamics; D. Cell division and chromosome partitioning; V. Defense mechanisms; T. Signal transduction mechanisms; M. Cell envelope biogenesis, outer membrane; N. Cell motility and secretion; Z. Cytoskeleton; U. Intracellular trafficking and secretion; O. Posttranslational modification, protein turnover, chaperones. C. Energy production and conversion; G. Carbohydrate transport and metabolism; E. Amino acid transport and metabolism; F. Nucleotide transport and metabolism; H. Coenzyme metabolism; I. Lipid metabolism; P. Inorganic ion transport and metabolism; Q. Secondary metabolites biosynthesis, transport and catabolism. R. General function prediction only; S. Function unknown. (-) : Not in COGs.
