# Supplementary material for: The prevalence of gene duplications and their ancient origin in Rhodobacter sphaeroides 2.4.1
Source: BMC Microbiol. 2010 Dec 30;10:331. doi: 10.1186/1471-2180-10-331 (PMC3024229; doi:10.1186/1471-2180-10-331)
Supplement: Additional file 2 — R. sphaeroides Ortholog Matches. This file contains detailed information about the highest ortholog matches of each of the proteins in a duplicate pair to bacteria outside of the R. sphaeroides species. [file 1471-2180-10-331-S2.PDF]

Supplementary Material
TABLE 2. *R. sphaeroides* Ortholog Matches

NO.	Orf 1[*]	Orf 2[*]	%I[†]	Orf 1 Highest Ortholog Match[‡]	%I[§]	Orf 2 Highest Ortholog Match[‡]	%I[§]	Tree Type[¶]	Bootstrap Value[#]
1	RSP_0036	RSP_1325	33	*Lawsonia intracellularis* PHE/MN1-00 (δ)	32	*Ruegeria pomeroyi* DSS-3 (α)	56	A	95
2	RSP_0047	RSP_1588	42	*Pelobacter carbinolicus* DSM 2380 (δ)	45	*Magnetococcus* sp. MC-1 (Other Bacteria)	52	A	100
3	RSP_0054	RSP_2220	24	*Thioalkalivibrio* sp. HL-EbGR7 (γ)	46	*Roseobacter denitrificans* OCh 114 (α)	55	A	100
4	RSP_0056	RSP_1332	39	*Enterobacter* sp. 638 (γ)	50	*Ruegeria* sp. TM1040 (α)	60	A	100
5	RSP_0061	RSP_1310	35	*Pseudomonas stutzeri* A1501 (γ)	58	*Ruegeria pomeroyi* DSS-3 (α)	65	A	100
6	RSP_0064	RSP_1328	45	*Burkholderia glumae* BGR1 (β)	53	*Paracoccus denitrificans* PD1222 (α)	70	A	100
7	RSP_0065	RSP_1321	34	*Thiomicrospira crunogena* XCL-2 (γ)	34	*Ruegeria pomeroyi* DSS-3 (α)	53	A	100
8	RSP_0074	RSP_1304	29	*Geobacter sulfurreducens* PCA (δ)	31	*Paracoccus denitrificans* PD1222 (α)	43	A	100
9	RSP_0077	RSP_1324	29	*Marinomonas* sp. MWYL1 (γ)	41	*Roseobacter denitrificans* OCh 114 (α)	68	A	100
10	RSP_0079	RSP_1327	29	*Halorhodospira halophila* SL1 (γ)	37	*Roseobacter denitrificans* OCh 114 (α)	57	A	100
11	RSP_0082	RSP_1330	28	*Sphingomonas wittichii* RW1 (α)	49	*Paracoccus denitrificans* PD1222 (α)	63	A	100
12	RSP_0083	RSP_1331	31	*Thioalkalivibrio* sp. HL-EbGR7 (γ)	55	*Ruegeria pomeroyi* DSS-3 (α)	46	A	100
13	RSP_0100	RSP_2512	36	*Rhodopseudomonas palustris* BisA53 (α)	45	*Roseobacter denitrificans* OCh 114 (α)	82	A	100
14	RSP_0102	RSP_2515	41	*Rhodopseudomonas palustris* BisB18 (α)	68	*Dinoroseobacter shibae* DFL 12 (α)	84	A	100
15	RSP_0104	RSP_2518	38	*Rhodopseudomonas palustris* BisA53 (α)	60	*Paracoccus denitrificans* PD1222 (α)	86	A	100
16	RSP_0105	RSP_2521	28	*Rhodopseudomonas palustris* BisA53 (α)	56	*Paracoccus denitrificans* PD1222 (α)	80	A	100
17	RSP_0108	RSP_2525	31	*Rhodopseudomonas palustris* CGA009 (α)	50	*Dinoroseobacter shibae* DFL 12 (α)	70	A	100
18	RSP_0112	RSP_2530	32	*Rhodopseudomonas palustris* BisB18 (α)	54	*Dinoroseobacter shibae* DFL 12 (α)	67	A	100
19	RSP_0146	RSP_0889	62	*Paracoccus denitrificans* PD1222 (α)	89	*Dinoroseobacter shibae* DFL 12 (α)	91	A	100
20	RSP_0153	RSP_2225	43	*Dinoroseobacter shibae* DFL 12 (α)	37	*Jannaschia* sp. CCS1 (α)	42	A	69
21	RSP_0161	RSP_1883	69	*Paracoccus denitrificans* PD1222 (α)	66	*Paracoccus denitrificans* PD1222 (α)	71	B	51
22	RSP_0176	RSP_3074	28	*Ruegeria pomeroyi* DSS-3 (α)	79	*Paracoccus denitrificans* PD1222 (α)	87	A	100
23	RSP_0180	RSP_3166	30	*Ruegeria* sp. TM1040 (α)	74	*Dinoroseobacter shibae* DFL 12 (α)	71	A	100
24	RSP_0183	RSP_3462	36	*Ruegeria* sp. TM1040 (α)	87	*Azorhizobium caulinodans* ORS 571 (α)	72	A	100
25	RSP_0189	RSP_1149	31	*Dinoroseobacter shibae* DFL 12 (α)	70	*Dinoroseobacter shibae* DFL 12 (α)	78	A	100
26	RSP_0224	RSP_0658	31	*Burkholderia phymatum* STM815 (β)	46	*Ruegeria pomeroyi* DSS-3 (α)	76	A	100
27	RSP_0229	RSP_2238	31	*Rhizobium leguminosarum* bv. viciae 3841 (α)	64	*Mesorhizobium* sp. BNC1 (α)	70	A	100
28	RSP_0235	RSP_3050	65	*Paracoccus denitrificans* PD1222 (α)	82	*Paracoccus denitrificans* PD1222 (α)	67	B	60
29	RSP_0248	RSP_0479	36	*Paracoccus denitrificans* PD1222 (α)	79	*Paracoccus denitrificans* PD1222 (α)	79	A	100
30	RSP_0254	RSP_1134	66	*Dinoroseobacter shibae* DFL 12 (α)	73	*Roseobacter denitrificans* OCh 114 (α)	79	A	100
31	RSP_0287	RSP_2827	27	*Roseobacter denitrificans* OCh 114 (α)	70	*Ruegeria* sp. TM1040 (α)	69	A	100
32	RSP_0314	RSP_1556	94	*Rhodopseudomonas palustris* HaA2 (α)	68	*Jannaschia* sp. CCS1 (α)	74	B	91
33	RSP_0329	RSP_3513	43	*Mesorhizobium loti* MAFF303099 (α)	40	*Mesorhizobium loti* MAFF303099 (α)	47	B	100
34	RSP_0345	RSP_3560	30	*Dinoroseobacter shibae* DFL 12 (α)	80	*Ruegeria pomeroyi* DSS-3 (α)	72	A	100
35	RSP_0382[*]	RSP_1257[*]	36	*Dinoroseobacter shibae* DFL 12 (α)	67	*Paracoccus denitrificans* PD1222 (α)	59	A	100
36	RSP_0413	RSP_4018	40	*Jannaschia* sp. CCS1 (α)	44	*Agrobacterium tumefaciens* str. C58 (α)	43	A	100
37	RSP_0423	RSP_3201	33	*Rhizobium leguminosarum* bv. trifolii WSM2304 (α)	56	*Methylobacterium extorquens* PA1 (α)	80	A	100
38	RSP_0476	RSP_2364	71	*Bradyrhizobium* japonicum USDA 110 (α)	65	*Marinomonas* sp. MWYL1 (γ)	62	B	96
39	RSP_0480	RSP_0096	41	*Agrobacterium vitis* S4 (α)	67	*Ochrobactrum anthropi* ATCC 49188 (α)	61	A	100
40	RSP_0563	RSP_2815	35	*Jannaschia* sp. CCS1 (α)	65	*Roseobacter denitrificans* OCh 114 (α)	71	A	100
41	RSP_0576	RSP_3142	30	*Paracoccus denitrificans* PD1222 (α)	73	*Xanthobacter autotrophicus* Py2 (α)	85	A	100
42	RSP_0578	RSP_1736	46	*Ruegeria pomeroyi* DSS-3 (α)	79	*Paracoccus denitrificans* PD1222 (α)	75	A	100
43	RSP_0601	RSP_2410	47	*Roseobacter denitrificans* OCh 114 (α)	80	*Ruegeria pomeroyi* DSS-3 (α)	84	A	100
44	RSP_0632	RSP_2155	38	*Ruegeria pomeroyi* DSS-3 (α)	80	*Sinorhizobium medicae* WSM419 (α)	79	A	100
45	RSP_0656	RSP_3330	64	*Ruegeria* sp. TM1040 (α)	63	*Jannaschia* sp. CCS1 (α)	59	B	100
46	RSP_0672	RSP_0920	40	*Roseobacter denitrificans* OCh 114 (α)	72	*Ochrobactrum anthropi* ATCC 49188 (α)	65	A	100
47	RSP_0688	RSP_0550	32	*Roseobacter denitrificans* OCh 114 (α)	55	*Paracoccus denitrificans* PD1222 (α)	62	A	100
48	RSP_0692	RSP_3027	67	*Ruegeria* sp. TM1040 (α)	72	*Dinoroseobacter shibae* DFL 12 (α)	67	B	89
49	RSP_0698	RSP_2572	35	*Ruegeria pomeroyi* DSS-3 (α)	77	*Mesorhizobium* sp. BNC1 (α)	46	A	100
50	RSP_0723	RSP_0240	31	*Ruegeria* sp. TM1040 (α)	81	*Agrobacterium tumefaciens* str. C58 (α)	81	A	100
51	RSP_0759	RSP_4086	29	*Ruegeria* sp. TM1040 (α)	63	*Vibrio vulnificus* CMCP6 (γ)	40	A	100
52	RSP_0766	RSP_0013	49	*Paracoccus denitrificans* PD1222 (α)	83	*Paracoccus denitrificans* PD1222 (α)	72	A	100
53	RSP_0772	RSP_1346	43	*Ruegeria pomeroyi* DSS-3 (α)	84	*Dinoroseobacter shibae* DFL 12 (α)	85	A	100
54	RSP_0817	RSP_2004	39	*Paracoccus denitrificans* PD1222 (α)	62	*Dinoroseobacter shibae* DFL 12 (α)	79	A	100
55	RSP_0840	RSP_1989	25	*Roseobacter denitrificans* OCh 114 (α)	74	*Dinoroseobacter shibae* DFL 12 (α)	59	A	100
56	RSP_0893	RSP_1120	28	*Dinoroseobacter shibae* DFL 12 (α)	57	*Ochrobactrum anthropi* ATCC 49188 (α)	34	A	100
57	RSP_0902[*]	RSP_0089	39	*Roseobacter denitrificans* OCh 114 (α)	54	*Shewanella frigidimarina* NCIMB 400 (γ)	51	A	100

58	RSP_0945	RSP_3035	30	*Brucella suis* 1330 (α)	67	*Mesorhizobium* sp. BNC1 (α)	57	A	100
59	RSP_0953	RSP_2415	30	*Mesorhizobium loti* MAFF303099 (α)	61	*Paracoccus denitrificans* PD1222 (α)	81	A	100
60	RSP_0961	RSP_2192	38	*Paracoccus denitrificans* PD1222 (α)	81	*Paracoccus denitrificans* PD1222 (α)	88	A	100
61	RSP_0970	RSP_1771	35	*Ruegeria pomeroyi* DSS-3 (α)	69	*Ruegeria pomeroyi* DSS-3 (α)	88	A	100
62	RSP_0971	RSP_2392	52	*Roseobacter denitrificans* OCh 114 (α)	53	*Roseobacter denitrificans* OCh 114 (α)	43	B	100
63	RSP_0976	RSP_3147	32	*Paracoccus denitrificans* PD1222 (α)	87	*Jannaschia* sp. CCS1 (α)	74	A	100
64	RSP_0979	RSP_3150	35	*Ruegeria* sp. TM1040 (α)	88	*Jannaschia* sp. CCS1 (α)	68	A	100
65	RSP_0990	RSP_3687	28	*Paracoccus denitrificans* PD1222 (α)	82	*Rhizobium leguminosarum* bv. viciae 3841 (α)	72	A	100
66	RSP_0992	RSP_3715	45	*Jannaschia* sp. CCS1 (α)	70	*Paracoccus denitrificans* PD1222 (α)	65	A	100
67	RSP_0993	RSP_3714	50	*Dinoroseobacter shibae* DFL 12 (α)	72	*Paracoccus denitrificans* PD1222 (α)	75	A	100
68	RSP_0994	RSP_3713	41	*Ruegeria pomeroyi* DSS-3 (α)	63	*Nitrosomonas europaea* ATCC 19718 (β)	54	A	100
69	RSP_1013	RSP_2814	28	*Agrobacterium tumefaciens* str. C58 (α)	63	*Dinoroseobacter shibae* DFL 12 (α)	66	A	100
70	RSP_1016	RSP_1572	50	*Ruegeria pomeroyi* DSS-3 (α)	79	*Dinoroseobacter shibae* DFL 12 (α)	72	A	100
71	RSP_1036	RSP_1035	30	*Ruegeria* sp. TM1040 (α)	75	*Paracoccus denitrificans* PD1222 (α)	59	A	100
72	RSP_1097	RSP_1832	30	*Dinoroseobacter shibae* DFL 12 (α)	65	*Jannaschia* sp. CCS1 (α)	70	A	100
73	RSP_1109	RSP_2147	36	*Dinoroseobacter shibae* DFL 12 (α)	77	*Paracoccus denitrificans* PD1222 (α)	83	A	100
74	RSP_1123	RSP_3553	30	*Dinoroseobacter shibae* DFL 12 (α)	70	*Roseobacter denitrificans* OCh 114 (α)	74	A	100
75	RSP_1184	RSP_2660	33	*Dinoroseobacter shibae* DFL 12 (α)	48	*Mesorhizobium* sp. BNC1 (α)	57	A	100
76	RSP_1255	RSP_2305	51	*Ruegeria pomeroyi* DSS-3 (α)	66	*Paracoccus denitrificans* PD1222 (α)	85	A	100
77	RSP_1260	RSP_6198	53	*Paracoccus denitrificans* PD1222 (α)	40	*Roseobacter denitrificans* OCh 114 (α)	35	B	100
78	RSP_1261	RSP_4060	61	*Rhodopseudomonas palustris* CGA009 (α)	42	*Bordetella avium* 197N (β)	44	B	100
79	RSP_1272	RSP_2681	48	*Paracoccus denitrificans* PD1222 (α)	74	*Mesorhizobium* sp. BNC1 (α)	55	B	100
80	RSP_1278	RSP_2504	39	*Paracoccus denitrificans* PD1222 (α)	50	*Roseobacter denitrificans* OCh 114 (α)	57	A	100
81	RSP_1282	RSP_3271	31	*Sinorhizobium medicae* WSM419 (α)	92	*Rhodopseudomonas palustris* HaA2 (α)	80	A	100
82	RSP_1283	RSP_3270	79	*Paracoccus denitrificans* PD1222 (α)	83	*Rhodopseudomonas palustris* BisB5 (α)	81	A	100
83	RSP_1284	RSP_3267	87	*Bradyrhizobium* sp. BTAi1 (α)	80	*Bradyrhizobium* sp. BTAi1 (α)	77	B	100
84	RSP_1285	RSP_3266	68	*Paracoccus denitrificans* PD1222 (α)	67	*Paracoccus denitrificans* PD1222 (α)	65	B	63
85	RSP_1303	RSP_0080	25	*Paracoccus denitrificans* PD1222 (α)	56	*Sphingomonas wittichii* RW1 (α)	38	A	100
86	RSP_1307	RSP_0076	43	*Ruegeria pomeroyi* DSS-3 (α)	75	*Thioalkalivibrio* sp. HL-EbGR7 (γ)	55	A	100
87	RSP_1309	RSP_0063	40	*Ruegeria* sp. TM1040 (α)	66	*Sphingomonas wittichii* RW1 (α)	65	A	100
88	RSP_1312	RSP_0053	30	*Ruegeria pomeroyi* DSS-3 (α)	54	*Sphingomonas wittichii* RW1 (α)	33	A	100
89	RSP_1319	RSP_0072	45	*Ruegeria pomeroyi* DSS-3 (α)	53	*Gluconobacter oxydans* 621H (α)	40	A	100
90	RSP_1320	RSP_0034	36	*Ruegeria pomeroyi* DSS-3 (α)	68	*Pseudomonas putida* F1 (γ)	49	A	100
91	RSP_1322	RSP_0066	33	*Ruegeria pomeroyi* DSS-3 (α)	55	*Sphingomonas wittichii* RW1 (α)	37	A	100
92	RSP_1326	RSP_0078	42	*Dinoroseobacter shibae* DFL 12 (α)	74	*Nitrosococcus oceani* ATCC 19707 (γ)	60	A	100
93	RSP_1379	RSP_2899	38	*Ruegeria pomeroyi* DSS-3 (α)	64	*Jannaschia* sp. CCS1 (α)	63	A	100
94	RSP_1463	RSP_3181	30	*Brucella melitensis* 16M (α)	63	*Bordetella parapertussis* 12822 (β)	62	A	100
95	RSP_1492	RSP_1869	35	*Ruegeria* sp. TM1040 (α)	65	*Ruegeria pomeroyi* DSS-3 (α)	68	A	100
96	RSP_1499	RSP_3272	29	*Ruegeria pomeroyi* DSS-3 (α)	70	*Ruegeria pomeroyi* DSS-3 (α)	73	A	100
97	RSP_1513	RSP_3585	45	*Thiomicrospira crunogena* XCL-2 (γ)	72	*Ruegeria* sp. TM1040 (α)	91	A	100
98	RSP_1532	RSP_0196	46	*Ruegeria pomeroyi* DSS-3 (α)	84	*Paracoccus denitrificans* PD1222 (α)	89	A	100
99	RSP_1551	RSP_0608	42	*Brucella melitensis* 16M (α)	49	*Mesorhizobium* sp. BNC1 (α)	46	A	100
100	RSP_1574	RSP_0820	35	*Ruegeria pomeroyi* DSS-3 (α)	41	*Rhizobium etli* CIAT 652 (α)	36	A	100
101	RSP_1591	RSP_3122	53	*Dinoroseobacter shibae* DFL 12 (α)	69	*Azorhizobium caulinodans* ORS 571 (α)	66	A	100
102	RSP_1613	RSP_1413	32	*Mesorhizobium* sp. BNC1 (α)	72	*Jannaschia* sp. CCS1 (α)	74	A	100
103	RSP_1614	RSP_1412	33	*Pseudomonas aeruginosa* UCBPP-PA14 (γ)	60	*Dinoroseobacter shibae* DFL 12 (α)	72	A	100
104	RSP_1638	RSP_2062	95	*Pelotomaculum thermopropionicum* SI (Firmicutes)	33	*Pelotomaculum thermopropionicum* SI (Firmicutes)	32	B	100
105	RSP_1645	RSP_3652	96	*Pseudomonas putida* KT2440 (γ)	42	*Pseudomonas putida* KT2440 (γ)	40	B	100
106	RSP_1647	RSP_3650	87	*Ruegeria* sp. TM1040 (α)	34	*Ruegeria* sp. TM1040 (α)	37	B	100
107	RSP_1650	RSP_2352	32	*Burkholderia cenocepacia* MC0-3 (β)	39	*Agrobacterium vitis* S4 (α)	53	A	100
108	RSP_1653	RSP_6190	82	*Nitrobacter hamburgensis* X14 (α)	31	*Neisseria gonorrhoeae* NCCP11945 (β)	40	B	100
109	RSP_1662	RSP_2080	39	*Paracoccus denitrificans* PD1222 (α)	47	*Paracoccus denitrificans* PD1222 (α)	42	A	100
110	RSP_1696	RSP_2630	39	*Roseobacter denitrificans* OCh 114 (α)	75	*Dinoroseobacter shibae* DFL 12 (α)	83	A	100
111	RSP_1706	RSP_1121	40	*Paracoccus denitrificans* PD1222 (α)	68	*Magnetospirillum magneticum* AMB-1 (α)	43	A	100
112	RSP_1766	RSP_1848	35	*Paracoccus denitrificans* PD1222 (α)	77	*Microcystis aeruginosa* NIES-843 (Cyanobacteria)	42	A	100
113	RSP_1767	RSP_2323	37	*Jannaschia* sp. CCS1 (α)	62	*Paracoccus denitrificans* PD1222 (α)	58	A	100
114	RSP_1787	RSP_2334	37	*Rhodospirillum rubrum* ATCC 11170 (α)	53	*Paracoccus denitrificans* PD1222 (α)	53	A	100
115	RSP_1820	RSP_0088	53	*Paracoccus denitrificans* PD1222 (α)	66	*Methylobacterium populi* BJ001 (α)	65	A	100
116	RSP_1843	RSP_1053	33	*Ruegeria* sp. TM1040 (α)	73	*Paracoccus denitrificans* PD1222 (α)	84	A	100
117	RSP_1850	RSP_1786	35	*Jannaschia* sp. CCS1 (α)	75	*Rhodospirillum rubrum* ATCC 11170 (α)	63	A	100
118	RSP_1889	RSP_3093	49	*Roseobacter denitrificans* OCh 114 (α)	72	*Agrobacterium radiobacter* K84 (α)	74	A	100

119	RSP_1894	RSP_2496	81	*Paracoccus denitrificans* PD1222 (α)	75	*Paracoccus denitrificans* PD1222 (α)	74	B	92
120	RSP_1927	RSP_3172	39	*Ruegeria pomeroyi* DSS-3 (α)	67	*Bradyrhizobium* japonicum USDA 110 (α)	48	A	100
121	RSP_1931	RSP_2653	31	*Ruegeria pomeroyi* DSS-3 (α)	82	*Ruegeria* sp. TM1040 (α)	83	A	100
122	RSP_1944	RSP_2820	42	*Roseobacter denitrificans* OCh 114 (α)	63	*Ruegeria pomeroyi* DSS-3 (α)	78	A	100
123	RSP_1951	RSP_3622	96	*Paracoccus denitrificans* PD1222 (α)	61	*Paracoccus denitrificans* PD1222 (α)	62	B	100
124	RSP_1954	RSP_6191	60	N/A		N/A		B	N.D.
125	RSP_1955	RSP_3647	72	N/A		*Pyrococcus furiosus* DSM 3638 (Euryarchaeota)	30	B	N.D.
126	RSP_1956	RSP_6196	92	*Ruegeria pomeroyi* DSS-3 (α)	50	*Ruegeria pomeroyi* DSS-3 (α)	51	B	100
127	RSP_1966	RSP_3007	99	*Desulfovibrio vulgaris* str. Hildenborough (δ)	46	*Aeromonas salmonicida* A449 (γ)	45	B	100
128	RSP_1984	RSP_2284	30	*Ruegeria* sp. TM1040 (α)	68	*Paracoccus denitrificans* PD1222 (α)	73	A	100
129	RSP_1998	RSP_3049	38	*Jannaschia* sp. CCS1 (α)	62	*Dinoroseobacter shibae* DFL 12 (α)	52	A	100
130	RSP_2061	RSP_1637	85	N/A		N/A		B	N.D.
131	RSP_2063	RSP_1639	91	*Agrobacterium vitis* S4 (α)	27	*Agrobacterium vitis* S4 (α)	34	B	100
132	RSP_2064	RSP_6012	89	*Agrobacterium vitis* S4 (α)	37	*Agrobacterium vitis* S4 (α)	37	B	100
133	RSP_2065	RSP_1640	39	*Sodalis glossinidius* str. 'morsitans' (γ)	32	*Agrobacterium vitis* S4 (α)	38	A	80
134	RSP_2098	RSP_2326	27	*Paracoccus denitrificans* PD1222 (α)	64	*Ruegeria* sp. TM1040 (α)	64	A	100
135	RSP_2106	RSP_2325	31	*Ruegeria pomeroyi* DSS-3 (α)	78	*Paracoccus denitrificans* PD1222 (α)	66	A	100
136	RSP_2122	RSP_3346	33	*Dinoroseobacter shibae* DFL 12 (α)	87	*Ruegeria* sp. TM1040 (α)	80	A	100
137	RSP_2124	RSP_3347	32	*Ruegeria* sp. TM1040 (α)	73	*Ruegeria* sp. TM1040 (α)	77	A	100
138	RSP_2184	RSP_3292	39	*Ruegeria* sp. TM1040 (α)	77	*Paracoccus denitrificans* PD1222 (α)	75	A	100
139	RSP_2189	RSP_2508	32	*Paracoccus denitrificans* PD1222 (α)	91	*Ruegeria pomeroyi* DSS-3 (α)	83	A	100
140	RSP_2201	RSP_2200	50	*Paracoccus denitrificans* PD1222 (α)	70	*Dinoroseobacter shibae* DFL 12 (α)	78	A	100
141	RSP_2227	RSP_2886	38	*Jannaschia* sp. CCS1 (α)	39	*Jannaschia* sp. CCS1 (α)	73	A	81
142	RSP_2232	RSP_1001	39	*Jannaschia* sp. CCS1 (α)	52	*Ruegeria* sp. TM1040 (α)	59	A	100
143	RSP_2247	RSP_1708	28	*Magnetospirillum magneticum* AMB-1 (α)	56	*Paracoccus denitrificans* PD1222 (α)	87	A	100
144	RSP_2297	RSP_3935	46	*Dinoroseobacter shibae* DFL 12 (α)	90	*Beijerinckia indica* ATCC 9039 (α)	63	A	100
145	RSP_2360	RSP_2997	29	*Mesorhizobium* sp. BNC1 (α)	46	*Sinorhizobium medicae* WSM419 (α)	30	A	100
146	RSP_2397	RSP_3040	32	*Ruegeria pomeroyi* DSS-3 (α)	63	*Ochrobactrum anthropi* ATCC 49188 (α)	64	A	100
147	RSP_2441	RSP_1589	26	*Rhodopseudomonas palustris* BisB18 (α)	23	*Bradyrhizobium* sp. BTAi1 (α)	26	B	100
148	RSP_2459	RSP_2809	34	*Ruegeria* sp. TM1040 (α)	73	*Dinoroseobacter shibae* DFL 12 (α)	56	A	100
149	RSP_2470	RSP_2996	51	*Ruegeria pomeroyi* DSS-3 (α)	64	*Jannaschia* sp. CCS1 (α)	50	A	85
150	RSP_2482	RSP_4189	94	*Dinoroseobacter shibae* DFL 12 (α)	87	*Dinoroseobacter shibae* DFL 12 (α)	87	B	100
151	RSP_2501	RSP_3873	46	*Magnetospirillum magneticum* AMB-1 (α)	46	*Magnetospirillum magneticum* AMB-1 (α)	55	A	57
152	RSP_2513	RSP_0101	53	*Ruegeria pomeroyi* DSS-3 (α)	91	*Rhodopseudomonas palustris* BisB18 (α)	69	A	100
153	RSP_2522	RSP_0106	43	*Dinoroseobacter shibae* DFL 12 (α)	83	*Rhodopseudomonas palustris* BisB5 (α)	72	A	100
154	RSP_2523	RSP_0107	43	*Ruegeria* sp. TM1040 (α)	90	*Rhodopseudomonas palustris* BisB5 (α)	69	A	100
155	RSP_2527	RSP_0110	34	*Ruegeria pomeroyi* DSS-3 (α)	81	*Rhodopseudomonas palustris* TIE-1 (α)	44	A	100
156	RSP_2565	RSP_1159	66	*Dinoroseobacter shibae* DFL 12 (α)	66	*Paracoccus denitrificans* PD1222 (α)	80	A	90
157	RSP_2592	RSP_2881	43	*Paracoccus denitrificans* PD1222 (α)	50	*Dinoroseobacter shibae* DFL 12 (α)	62	A	100
158	RSP_2607	RSP_3017	42	*Agrobacterium tumefaciens* str. C58 (α)	64	*Pseudomonas entomophila* L48 (γ)	62	A	100
159	RSP_2618	RSP_3826	35	*Dinoroseobacter shibae* DFL 12 (α)	65	*Paracoccus denitrificans* PD1222 (α)	68	A	100
160	RSP_2623	RSP_2850	35	*Ruegeria pomeroyi* DSS-3 (α)	74	*Ruegeria pomeroyi* DSS-3 (α)	74	A	100
161	RSP_2673	RSP_2578	25	*Agrobacterium tumefaciens* str. C58 (α)	69	*Sinorhizobium meliloti* 1021 (α)	79	A	100
162	RSP_2749	RSP_0217	27	*Polaromonas* sp. JS666 (β)	47	*Rhizobium* sp. NGR234 (α)	47	A	100
163	RSP_2779	RSP_2380	42	*Saccharophagus degradans* 2-40 (γ)	76	*Nitrosococcus oceani* ATCC 19707 (γ)	66	A	100
164	RSP_2781	RSP_0899	36	*Ruegeria pomeroyi* DSS-3 (α)	76	*Ruegeria* sp. TM1040 (α)	73	A	100
165	RSP_2846	RSP_0154	36	*Ruegeria* sp. TM1040 (α)	77	*Ruegeria* sp. TM1040 (α)	79	A	100
166	RSP_2856	RSP_0704	39	*Dinoroseobacter shibae* DFL 12 (α)	62	*Dinoroseobacter shibae* DFL 12 (α)	69	A	100
167	RSP_2888	RSP_3341	38	*Dinoroseobacter shibae* DFL 12 (α)	61	*Oligotropha carboxidovorans* OM5 (α)	54	A	100
168	RSP_2907	RSP_2977	42	*Dinoroseobacter shibae* DFL 12 (α)	79	*Paracoccus denitrificans* PD1222 (α)	83	A	100
169	RSP_2909	RSP_3743	40	*Ruegeria pomeroyi* DSS-3 (α)	66	*Rubrobacter xylanophilus* DSM 9941 (Actinobacteria)	53	A	100
170	RSP_3015	RSP_3969	56	*Burkholderia xenovorans* LB400 (β)	66	*Mesorhizobium loti* MAFF303099 (α)	76	A	100
171	RSP_3028	RSP_2984	54	*Methylobacterium extorquens* AM1 (α)	66	*Jannaschia* sp. CCS1 (α)	77	A	100
172	RSP_3046	RSP_4118	48	*Rhodospirillum rubrum* ATCC 11170 (α)	53	*Agrobacterium tumefaciens* str. C58 (α)	80	A	100
173	RSP_3098	RSP_0118	33	*Rhodopseudomonas palustris* HaA2 (α)	55	*Nitrosococcus oceani* ATCC 19707 (γ)	46	A	100
174	RSP_3106	RSP_4276	32	*Sinorhizobium meliloti* 1021 (α)	67	*Shewanella piezotolerans* WP3 (γ)	65	A	100
175	RSP_3113	RSP_2316	28	*Agrobacterium tumefaciens* str. C58 (α)	69	*Burkholderia vietnamiensis* G4 (β)	40	A	100
176	RSP_3116	RSP_3345	31	*Mesorhizobium* sp. BNC1 (α)	49	*Paracoccus denitrificans* PD1222 (α)	50	A	100
177	RSP_3134	RSP_3475	34	*Methylocella silvestris* BL2 (α)	41	*Rhizobium etli* CIAT 652 (α)	44	A	100
178	RSP_3144	RSP_4010	52	*Caulobacter* sp. K31 (α)	53	*Paracoccus denitrificans* PD1222 (α)	82	A	100
179	RSP_3187	RSP_0125	43	*Ralstonia eutropha* JMP134 (β)	50	*Mesorhizobium* sp. BNC1 (α)	53	A	100

180	RSP_3253	RSP_3443	38	*Sinorhizobium meliloti* 1021 (α)	75	*Paracoccus denitrificans* PD1222 (α)	67	A	100
181	RSP_3268	RSP_2956	58	*Paracoccus denitrificans* PD1222 (α)	73	*Jannaschia* sp. CCS1 (α)	71	A	100
182	RSP_3275	RSP_1024	45	*Paracoccus denitrificans* PD1222 (α)	71	*Ruegeria pomeroyi* DSS-3 (α)	77	A	100
183	RSP_3288	RSP_1444	31	*Paracoccus denitrificans* PD1222 (α)	79	*Mesorhizobium* sp. BNC1 (α)	84	A	100
184	RSP_3325	RSP_3719	58	*Mesorhizobium loti* MAFF303099 (α)	53	*Mesorhizobium loti* MAFF303099 (α)	65	B	92
185	RSP_3342	RSP_1546	84	*Paracoccus denitrificans* PD1222 (α)	83	*Paracoccus denitrificans* PD1222 (α)	79	B	96
186	RSP_3349	RSP_4072	64	*Anaeromyxobacter dehalogenans* 2CP-1 (δ)	29	*Campylobacter lari* RM2100 (ε)	33	B	99
187	RSP_3350	RSP_4071	64	*Ruegeria* sp. TM1040 (α)	22	*Ruegeria* sp. TM1040 (α)	22	B	100
188	RSP_3372	RSP_0097	32	*Rhizobium leguminosarum* bv. viciae 3841 (α)	77	*Mesorhizobium loti* MAFF303099 (α)	81	A	100
189	RSP_3397	RSP_3515	29	*Verminephrobacter eiseniae* EF01-2 (β)	71	*Geobacillus thermodenitrificans* NG80-2 (Firmicutes)	32	A	100
190	RSP_3398	RSP_3657	37	*Verminephrobacter eiseniae* EF01-2 (β)	80	*Gluconacetobacter diazotrophicus* PAl 5 (α)	47	A	100
191	RSP_3406	RSP_2904	39	*Sinorhizobium meliloti* 1021 (α)	65	*Jannaschia* sp. CCS1 (α)	67	A	100
192	RSP_3410	RSP_3386	30	*Escherichia coli* CFT073 (γ)	36	*Chromohalobacter salexigens* DSM 3043 (γ)	55	A	100
193	RSP_3436	RSP_6165	37	*Paracoccus denitrificans* PD1222 (α)	79	*Jannaschia* sp. CCS1 (α)	50	A	100
194	RSP_3497	RSP_4069	31	*Bradyrhizobium japonicum* USDA 110 (α)	38	*Bradyrhizobium* sp. ORS278 (α)	48	A	100
195	RSP_3505	RSP_0090	31	*Sinorhizobium meliloti* 1021 (α)	57	*Pseudoalteromonas haloplanktis* TAC125 (γ)	49	A	100
196	RSP_3547	RSP_2495	31	*Paracoccus denitrificans* PD1222 (α)	82	*Paracoccus denitrificans* PD1222 (α)	85	A	100
197	RSP_3574	RSP_2935	36	*Halorhodospira halophila* SL1 (γ)	43	*Ruegeria pomeroyi* DSS-3 (α)	77	A	100
198	RSP_3608	RSP_4107	32	*Mesorhizobium* sp. BNC1 (α)	45	*Gluconacetobacter diazotrophicus* PAl 5 (α)	35	A	100
199	RSP_3624	RSP_3792	92	*Paracoccus denitrificans* PD1222 (α)	70	*Paracoccus denitrificans* PD1222 (α)		B	100
200	RSP_3627	RSP_3784	95	*Agrobacterium vitis* S4 (α)	43	*Agrobacterium vitis* S4 (α)	44	B	100
201	RSP_3628	RSP_3786	84	N/A		N/A		B	N.D.
202	RSP_3664	RSP_3405	34	*Rhizobium* sp. NGR234 (α)	39	*Methylobacterium nodulans* ORS 2060 (α)	59	A	100
203	RSP_3695	RSP_0237	31	*Klebsiella pneumoniae* 342 (γ)	47	*Ruegeria pomeroyi* DSS-3 (α)	66	A	100
204	RSP_3697	RSP_3861	47	*Paracoccus denitrificans* PD1222 (α)	73	*Paracoccus denitrificans* PD1222 (α)	78	A	100
205	RSP_3703	RSP_1179	31	*Klebsiella pneumoniae* NTUH-K2044 (γ)	72	*Dinoroseobacter shibae* DFL 12 (α)	75	A	100
206	RSP_3843	RSP_0024	39	*Ruegeria* sp. TM1040 (α)	64	*Chloroflexus aggregans* DSM 9485 (Chloroflexi)	62	A	100
207	RSP_3884	RSP_3887	31	*Beutenbergia cavernae* DSM 12333 (Actinobacteria)	67	*Saccharopolyspora erythraea* NRRL 2338 (Actinobacteria)	61	A	100
208	RSP_3894	RSP_3978	96	*Agrobacterium tumefaciens* str. C58 (α)	57	*Agrobacterium tumefaciens* str. C58 (α)	58	B	100
209	RSP_3904	RSP_7352	98	*Salmonella enterica* serovar Dublin (γ)	34	*Nostoc punctiforme* PCC 73102 (Cyanobacteria)	41	B	100
210	RSP_3906	RSP_2981	35	*Ruegeria pomeroyi* DSS-3 (α)	36	*Ruegeria pomeroyi* DSS-3 (α)	52	B	70
211	RSP_3908	RSP_4251	99	*Jannaschia* sp. CCS1 (α)	44	*Jannaschia* sp. CCS1 (α)	44	B	100
212	RSP_3955	RSP_0726	36	*Polaromonas* sp. JS666 (β)	46	*Paracoccus denitrificans* PD1222 (α)	80	A	100
213	RSP_3956	RSP_3940	35	*Sinorhizobium medicae* WSM419 (α)	67	*Rhizobium* sp. NGR234 (α)	38	B	99
214	RSP_3985	RSP_3920	46	*Agrobacterium vitis* S4 (α)	46	*Agrobacterium vitis* S4 (α)	47	B	100
215	RSP_4008	RSP_2813	42	*Mesorhizobium loti* MAFF303099 (α)	53	*Roseobacter denitrificans* OCh 114 (α)	84	A	100
216	RSP_4021	RSP_7387	39	*Rhizobium leguminosarum* bv. viciae 3841 (α)	44	*Dinoroseobacter shibae* DFL 12 (α)	82	A	100
217	RSP_4022	RSP_4185	35	*Agrobacterium vitis* S4 (α)	40	*Dinoroseobacter shibae* DFL 12 (α)	55	A	100
218	RSP_4050	RSP_4049	66	*Paracoccus denitrificans* PD1222 (α)	73	*Jannaschia* sp. CCS1 (α)	77	A	100
219	RSP_4053	RSP_0834	67	*Roseobacter denitrificans* OCh 114 (α)	69	*Ruegeria* sp. TM1040 (α)	66	A	49
220	RSP_4103	RSP_1428	41	*Nostoc* sp. PCC 7120 (Cyanobacteria)	28	*Clostridium botulinum F* str. Langeland (Firmicutes)	29	B	100
221	RSP_4138	RSP_3902	96	*Ruegeria* sp. TM1040 (α)	47	*Ruegeria* sp. TM1040 (α)	47	B	100
222	RSP_4139	RSP_3901	61	*Roseobacter denitrificans* OCh 114 (α)	25	*Sodalis glossinidius* str. 'morsitans' (γ)	28	B	99
223	RSP_4165	RSP_4182	43	*Rhizobium leguminosarum* bv. trifolii WSM1325 (α)	51	*Agrobacterium vitis* S4 (α)	73	A	100
224	RSP_4178	RSP_3012	74	*Agrobacterium vitis* S4 (α)	47	*Sinorhizobium meliloti* 1021 (α)	57	B	100
225	RSP_4207	RSP_3721	66	*Mesorhizobium loti* MAFF303099 (α)	47	*Rhizobium* sp. NGR234 (α)	50	B	100
226	RSP_4209	RSP_3723	71	*Bradyrhizobium* sp. ORS278 (α)	33	*Bradyrhizobium* sp. ORS278 (α)	29	B	100
227	RSP_4252	RSP_3907	98	N/A		N/A		B	N.D.
228	RSP_6015	RSP_6036	65	N/A		*Paracoccus denitrificans* PD1222 (α)	59	B	N.D.
229	RSP_6035	RSP_3772	64	*Paracoccus denitrificans* PD1222 (α)	32	N/A		B	N.D.
230	RSP_6194	RSP_6200	96	N/A		N/A		B	N.D.
231	RSP_6234	RSP_6250	39	*Caulobacter* sp. K31 (α)	32	*Caulobacter* sp. K31 (α)	37	A	67
232	RSP_6256	RSP_6158	58	*Bradyrhizobium* sp. BTAi1 (α)	47	*Oligotropha carboxidovorans* OM5 (α)	37	B	66
233	RSP_7246	RSP_4028	67	*Anabaena variabilis* ATCC 29413 (Cyanobacteria)	30	*Anabaena variabilis* ATCC 29413 (Cyanobacteria)	35	B	100
234	RSP_7390	RSP_3896	85	*Rhizobium etli* CFN 42 (α)	26	*Rhizobium etli* CIAT 652 (α)	28	B	100

[*] The protein coding genes in a duplicate gene pair
[†] The amino acid identity between the protein coding genes in the gene pair
[‡] The highest ortholog match for the corresponding Orf. The group that the ortholog is present in is given in parenthesis to its right. The group in Proteobacteria are abbreviated according to their subdivion. N/A represents no ortholog match was found.
[§] The amino acid identity between the Orf and its highest matching ortholog.
[¶] The tree type (Type-A or Type-B).
[#] The bootstrap value for the given tree. N.D. stands for not determined.
