# Supplementary material for: The prevalence of gene duplications and their ancient origin in Rhodobacter sphaeroides 2.4.1
Source: BMC Microbiol. 2010 Dec 30;10:331. doi: 10.1186/1471-2180-10-331 (PMC3024229; doi:10.1186/1471-2180-10-331)
Supplement: Additional file 3 — R. sphaeroides Strain Hits. This file contains information concerning the number of hits of a protein in a duplicate pair in R. sphaeroides 2.4.1 to three other R. sphaeroides strains (ATCC 17025, ATCC 17029, and KD131). [file 1471-2180-10-331-S3.PDF]

Supplementary Material
TABLE 3. *R. sphaeroides* Strain Hits

**NO.**	***R. sphaeroides* 2.4.1 Orf 1***	**# *R. sphaeroides* ATCC 17025 Hits†**	**# *R. sphaeroides* ATCC 17029 Hits†**	**# *R. sphaeroides* KD131 Hits†**
1	RSP_0036	1	1	1
2	RSP_0047	2	1	2
3	RSP_0054	1	0	0
4	RSP_0056	1	4	4
5	RSP_0061	1	1	0
6	RSP_0064	1	1	1
7	RSP_0065	1	0	1
8	RSP_0074	1	1	1
9	RSP_0077	1	1	1
10	RSP_0079	1	1	1
11	RSP_0082	1	1	1
12	RSP_0083	1	1	1
13	RSP_0100	1	0	0
14	RSP_0102	2	2	1
15	RSP_0104	3	3	2
16	RSP_0105	1	2	1
17	RSP_0108	1	1	1
18	RSP_0112	1	1	1
19	RSP_0146	2	0	0
20	RSP_0153	2	1	1
21	RSP_0161	2	1	3
22	RSP_0176	2	3	3
23	RSP_0180	2	1	1
24	RSP_0183	2	2	2
25	RSP_0189	2	2	2
26	RSP_0224	1	1	1
27	RSP_0229	1	2	2
28	RSP_0235	1	1	1
29	RSP_0248	2	0	1
30	RSP_0254	2	3	2
31	RSP_0287	1	1	1
32	RSP_0314	1	0	0
33	RSP_0329	2	1	2
34	RSP_0345	2	2	1
35	RSP_0382	2	2	2
36	RSP_0413	2	3	4
37	RSP_0423	1	1	2
38	RSP_0476	1	1	2
39	RSP_0480	2	3	3
40	RSP_0563	2	1	2
41	RSP_0576	1	1	2
42	RSP_0578	2	1	2
43	RSP_0601	3	0	1
44	RSP_0632	1	2	2
45	RSP_0656	2	1	2

46	RSP_0672	2	1	1
47	RSP_0688	2	2	2
48	RSP_0692	1	2	2
49	RSP_0698	2	0	1
50	RSP_0723	2	2	2
51	RSP_0759	2	1	1
52	RSP_0766	2	2	2
53	RSP_0772	2	1	2
54	RSP_0817	2	1	2
55	RSP_0840	1	1	1
56	RSP_0893	1	0	0
57	RSP_0902	3	2	1
58	RSP_0945	1	2	2
59	RSP_0953	2	2	2
60	RSP_0961	2	3	3
61	RSP_0970	2	2	2
62	RSP_0971	2	1	2
63	RSP_0976	1	1	2
64	RSP_0979	1	0	0
65	RSP_0990	1	1	1
66	RSP_0992	4	4	4
67	RSP_0993	2	0	1
68	RSP_0994	2	2	2
69	RSP_1013	1	1	1
70	RSP_1016	2	1	2
71	RSP_1036	1	0	0
72	RSP_1097	1	1	1
73	RSP_1109	2	0	1
74	RSP_1123	0	2	2
75	RSP_1184	2	0	1
76	RSP_1255	2	1	1
77	RSP_1260	2	0	1
78	RSP_1261	1	1	1
79	RSP_1272	2	0	1
80	RSP_1278	2	2	1
81	RSP_1282	3	1	0
82	RSP_1283	2	2	2
83	RSP_1284	2	0	1
84	RSP_1285	2	2	2
85	RSP_1303	1	0	0
86	RSP_1307	2	2	1
87	RSP_1309	2	2	2
88	RSP_1312	2	1	2
89	RSP_1319	2	1	2
90	RSP_1320	2	2	2
91	RSP_1322	2	2	2
92	RSP_1326	2	2	2
93	RSP_1379	2	2	2

94	RSP_1463	2	2	4
95	RSP_1492	2	2	2
96	RSP_1499	1	1	1
97	RSP_1513	2	0	2
98	RSP_1532	2	0	0
99	RSP_1551	0	0	1
100	RSP_1574	0	1	1
101	RSP_1591	1	2	1
102	RSP_1613	3	1	0
103	RSP_1614	1	3	3
104	RSP_1638	0	1	2
105	RSP_1645	1	0	2
106	RSP_1647	2	0	3
107	RSP_1650	0	2	1
108	RSP_1653	0	0	5
109	RSP_1662	0	0	2
110	RSP_1696	2	2	2
111	RSP_1706	0	2	2
112	RSP_1766	1	2	2
113	RSP_1767	2	1	2
114	RSP_1787	1	1	2
115	RSP_1820	2	1	2
116	RSP_1843	2	2	2
117	RSP_1850	2	2	1
118	RSP_1889	2	0	1
119	RSP_1894	1	1	0
120	RSP_1927	1	2	2
121	RSP_1931	2	1	1
122	RSP_1944	2	1	1
123	RSP_1951	1	1	1
124	RSP_1954	1	1	0
125	RSP_1955	0	1	0
126	RSP_1956	1	1	1
127	RSP_1966	0	2	0
128	RSP_1984	1	2	2
129	RSP_1998	1	2	2
130	RSP_2061	0	0	0
131	RSP_2063	0	1	1
132	RSP_2064	0	2	2
133	RSP_2065	0	2	2
134	RSP_2098	1	0	1
135	RSP_2106	1	0	1
136	RSP_2122	2	1	1
137	RSP_2124	2	1	2
138	RSP_2184	2	1	2
139	RSP_2189	2	1	2
140	RSP_2201	2	1	0
141	RSP_2227	2	1	2

142	RSP_2232	2	1	1
143	RSP_2247	1	1	1
144	RSP_2297	1	1	1
145	RSP_2360	2	1	0
146	RSP_2397	1	2	2
147	RSP_2441	1	1	1
148	RSP_2459	2	1	2
149	RSP_2470	1	1	1
150	RSP_2482	2	1	0
151	RSP_2501	1	2	2
152	RSP_2513	2	2	0
153	RSP_2522	2	2	1
154	RSP_2523	2	1	0
155	RSP_2527	4	4	4
156	RSP_2565	2	1	1
157	RSP_2592	1	2	2
158	RSP_2607	0	0	0
159	RSP_2618	2	2	2
160	RSP_2623	2	3	3
161	RSP_2673	2	2	2
162	RSP_2749	0	0	0
163	RSP_2779	1	2	2
164	RSP_2781	0	1	2
165	RSP_2846	2	2	2
166	RSP_2856	2	2	2
167	RSP_2888	2	0	0
168	RSP_2907	2	2	2
169	RSP_2909	1	2	2
170	RSP_3015	2	1	1
171	RSP_3028	2	3	3
172	RSP_3046	3	1	1
173	RSP_3098	2	2	2
174	RSP_3106	0	1	1
175	RSP_3113	1	1	1
176	RSP_3116	2	2	2
177	RSP_3134	1	2	1
178	RSP_3144	0	1	1
179	RSP_3187	2	1	2
180	RSP_3253	2	1	2
181	RSP_3268	2	3	3
182	RSP_3275	1	1	2
183	RSP_3288	1	1	1
184	RSP_3325	2	2	2
185	RSP_3342	2	2	0
186	RSP_3349	1	1	1
187	RSP_3350	1	1	1
188	RSP_3372	3	3	2
189	RSP_3397	0	1	1

190	RSP_3398	0	2	1
191	RSP_3406	1	2	1
192	RSP_3410	0	1	1
193	RSP_3436	1	0	0
194	RSP_3497	0	1	1
195	RSP_3505	1	2	2
196	RSP_3547	2	2	2
197	RSP_3574	0	2	2
198	RSP_3608	1	1	1
199	RSP_3624	0	1	1
200	RSP_3627	1	1	2
201	RSP_3628	0	1	1
202	RSP_3664	0	1	2
203	RSP_3695	1	3	2
204	RSP_3697	3	2	1
205	RSP_3703	1	3	4
206	RSP_3843	1	1	1
207	RSP_3884	0	2	0
208	RSP_3894	0	0	1
209	RSP_3904	0	0	0
210	RSP_3906	0	1	1
211	RSP_3908	0	0	0
212	RSP_3955	2	1	2
213	RSP_3956	2	0	1
214	RSP_3985	1	1	1
215	RSP_4008	1	0	2
216	RSP_4021	0	0	1
217	RSP_4022	0	0	1
218	RSP_4050	3	3	3
219	RSP_4053	1	1	1
220	RSP_4103	0	0	0
221	RSP_4138	0	0	0
222	RSP_4139	0	0	0
223	RSP_4165	10	1	7
224	RSP_4178	2	0	0
225	RSP_4207	1	0	0
226	RSP_4209	1	1	1
227	RSP_4252	0	0	0
228	RSP_6015	0	0	0
229	RSP_6035	1	2	1
230	RSP_6194	0	1	1
231	RSP_6234	0	1	0
232	RSP_6256	1	0	0
233	RSP_7246	0	0	1
234	RSP_7390	1	0	0

* The query Orf from R. *sphaeroides* 2.4.1

† The number of hits in the *R. sphaeroides* strain meeting the designated criteria specified in the Materials and Methods section
